# Losartan in the Era of Emerging Contaminants: A Multi-Criteria Approach for Efficient and Sustainable Remediation

**DOI:** 10.3390/molecules31101746

**Published:** 2026-05-20

**Authors:** Jordana Georgin, Younes Dehmani, Noureddine El Messoaudi, Dison S. P. Franco

**Affiliations:** 1 Department of Civil and Environmental, Universidad de La Costa, CUC, Calle 58 # 55–66, Barranquilla 080002, Atlántico, Colombia; francodison@gmail.com; 2 Laboratory of Chemistry/Biology Applied to the Environment, Faculty of Sciences, Moulay Ismaïl University, BP 11201-Zitoune, Meknes 50070, Morocco; dehmaniy@gmail.com; 3 Laboratory of Computer Science and Interdisciplinary Physics (LIPI), ENS-Fez, Sidi Mohamed Ben Abdellah University, Bensouda, Fez P.O. Box 5206, Morocco; 4 Department of Chemistry, College of Science, Imam Mohammad Ibn Saud Islamic University (IMSIU), Riyadh 11623, Saudi Arabia

**Keywords:** emergent contaminants, catalysis, degradation, water treatment

## Abstract

This paper systematically reviews losartan, a hypertension pharmaceutical compound that is one of many newly identified emerging contaminants in water. Worldwide use of pharmaceuticals continues to grow, and losartan has been identified as a contaminant that frequently accumulates in aquatic systems as a result of this global increase in use. The paper presents systematic reviews on the environmental occurrence, physicochemical characteristics, analytical methods of detection, and remediation techniques associated with losartan contamination. Losartan is often detected at levels of ng L^−1^–µg L^−1^ in wastewater systems, surface water and marine ecosystems, very effectively demonstrating the inadequacies of existing conventional wastewater treatment facilities, which are typically capable of removing only 20–70% of the contamination, with this variability largely attributed to differences in hydraulic/solids retention times, operational conditions, influent organic load, and the limited microbial acclimatization to recalcitrant pharmaceutical compounds. Emerging remediation technologies demonstrate the potential for removal efficiencies of >90% include hybrid systems, advanced electrochemical processes, new improved adsorption systems, and novel material for adsorption. However, there are still considerable barriers to progress, including excessive energy use, high operating costs, and perhaps most concerning, potentially toxic transition products generated by partial degradation. Furthermore, the literature review identified key literature gaps: lack of specific regulations, absence of full-scale studies, and inconsistencies in by-product toxicity assessments. The conclusion of this review is that to achieve worldwide water security and sustainability of aquatic resources, effective mitigation of the environmental risks associated with losartan requires combined approaches comprising innovative technologies, comprehensive ecotoxicological investigations, and improved collaboration between scientists, policymakers, and industry.

## 1. Introduction

The presence of pharmaceutical compounds in aquatic ecosystems has become a major issue in environmental chemistry, sanitary engineering, and ecotoxicology over the last few decades [1]. As emerging contaminants, they have been described as such due to an increase in their presence throughout numerous environmental matrices as well as the lack of specific regulations in many countries. The increasing rate of drug use around the world, their incomplete removal through metabolic processes in humans or animals, and the inefficiency of most traditional WWTPs in removing micro-pollutants are all associated with the increasing number of detections of these compounds within aquatic systems [2,3,4]. Due to this fact, a variety of drugs have been identified within wastewater, surface water, and groundwater at concentrations that range from ng L^−1^ to µg L^−1^. This has raised concerns regarding potential ecotoxicological impacts and also impacts upon water quality [5].

Antihypertensive pharmaceuticals have become heavily scrutinized as the result of the increased number of people suffering from cardiac illnesses around the globe and the increased use of these types of medications as a consequence [6]. One of the most well-known antihypertensive medications in this group is Losartan an ARB. Losartan has been widely prescribed for the treatment of heart failure, diabetic nephropathy and hypertension. It has been estimated that approximately 52 million Losartan prescriptions were written for patients in the US in just one year, highlighting this medication’s widespread use clinically and therapeutic value for the treatment of diseases associated with the cardiovascular system [7]. Losartan is continuously discharged into the environment due to its extensive use and consumption. A significant fraction of losartan is excreted unchanged or as metabolites via urine and feces, entering municipal wastewater systems. Because biological treatment methods were not sufficiently performing by traditional means, the chemicals associated with losartan will frequently be present in the wastewater treatment processes. Losartan and its metabolites have, in turn, been discovered in a variety of surface bodies of water, coastal ecosystems, agricultural irrigation water, and as influents and effluents within wastewater treatment plants [8].

Environmental monitoring studies have demonstrated that this chemical can be found repeatedly across all of the different parts of the environment. The concentration of losartan in the effluent from wastewater treatment plants can be greater than 800 ng L^−1^, which indicates that this chemical can be highly persistent after it has undergone conventional treatment [9]. There are also studies that have been completed in river basins in Europe that have shown that the concentration of losartan in recycled water effluents is approximately 957 ng L^−1^, thus demonstrating that this chemical can also be potentially transported into aquatic ecosystems [10]. The concentration of the chemical in surface waters generally ranges from about 0.17 to 36.5 ng L^−1^, whereas in treated wastewater it can be as high as µg L^−1^ depending on the amount of use in the area and the efficiency of wastewater treatment [5]. Environmental contamination by losartan seriously affects ecotoxicology. Pharmaceutical pollutants can affect aquatic biological activity at low concentrations due to prolonged exposure and by disrupting the genetic progress of these organisms. Many drugs damage the metabolic processes of organisms at every unit in a food chain, such as vertebrates, phytoplankton and macroinvertebrate [11]. Furthermore, multiple drug contaminants in aquatic environments may exhibit additive or synergistic effects, thereby increasing the environmental risk of exposure to pharmaceutical contaminants [5].

The environmental presence of losartan is made even more complicated by the formation of transformation products during the chemical or biological degradation of the compound, which may lead to the formation of persistent metabolites and byproducts in wastewater treatment or natural environments [12]. The potential for transformation products to have toxicities equal to or greater than the parent compound complicates the assessment of environmental risk and the development of effective treatment programs [7]. In response to this situation, many technologies have been studied for their ability to remove pharmaceutical contaminants from environmental matrices and wastewater. Notable technologies studied include advanced oxidation processes, electrochemical systems, advanced adsorbents, and photocatalytic processes [13,14,15]. These technologies have been shown to be highly effective in degrading persistent organic micropollutants, such as drugs like losartan, which are commonly used. For example, electrochemical oxidation techniques have effectively removed a variety of pharmaceutical residues from municipal wastewater, demonstrating their application for treating emerging micropollutants [9].

There are still a lot of gaps in the scientific literature, especially when it comes to the comprehensive assessment of remediation techniques that are specifically used with losartan, even if the number of studies on the incidence and management of pharmaceutical pollutants is increasing. Without providing a thorough relative analysis of the effectiveness of versatile technologies, the degradation mechanisms involved, and the challenges associated with their implementation at full scale, many studies centralize on environmental detection or the isolated valuation of appendage of intervention. Therefore, a review of losartan’s environmental behavior and remediation techniques is essential. With an emphasis on the elementary redress techniques canvas, their operational constraints, and future prospects for the initiation of more effectual and sustainable strategy for the control of emerging pharmaceutic contamination, this review critically synthesizes recent advances regarding the environmental occurrence, fate, and remediation technologies for this pharmaceutical in aquatic systems.

## 2. Methodology

The intent of this paper is to systematically review the scientific literature on the occurrence, environmental fate, and remediation methods involving the drug Losartan (Cozaar) in aquatic environments. It was conducted with a critical evaluation protocol to gather together and assess the state of the art for mitigation methods of this emerging contaminant. The search of scientific papers over the past 15 years through four databases—Scopus, Web of Science, and ScienceDirect, in addition to PubMed—included publications using the keyword combinations Losartan, Environmental Occurrence, Wastewater Treatment, Advanced Oxidation, Adsorption, Ecotoxicology, and Remediation Technology. Original research articles, systematic reviews, and technical reports were the types of publications that the author focused on to provide quantitative data pertaining to environmental concentrations, removal efficiency, and ecotoxicological risk assessment to complete the search for literature reviewed in this paper.

Research studies must meet four specifications: i. focused upon losartan/a metabolite (such as EXP-3174); ii. conducted in water matrices (treated, treated sewage, surface water or ocean water); iii. containing validated analysis methods (for example LC-MS/MS); and iv. including conventional and/or advanced treatment technologies. Studies that exclusively considered human biological matrices and were not related to environmental matrices or studies lacking detailed experimental data were not included in this systematic review. A complete set of physicochemical properties (or characteristics), reported global concentrations of losartan/a metabolite within the environment, operational parameters for each treatment technology (doses of oxidants used, types of adsorbents used, residence times) and performance indicators (losartan and/or metabolite removal or total mineralization) for each treatment technology were prepared as a component of the systematic review data extraction. The data were organized in comparison charts to allow for critical assessments of the advantages and disadvantages of individual treatment technology methods.

Finally, a qualitative assessment of scientific information as it relates to the characterization of transformation products, the adoption of ecotoxicological criteria and the discrepancy between bench-scale and/or laboratory studies versus actual full-scale operational conditions was completed. In order to provide a foundation for future policy recommendations and technology scaling, this review attempts to correlate the technical efficiency of remediation techniques with their economic feasibility and environmental sustainability. We acknowledge that the current absence of specific regulatory guidelines for losartan, along with variability in analytical LOQs across different laboratories, limits the immediate operational applicability of such multi-criteria comparisons. Therefore, this analysis is intentionally prospective, serving as a strategic framework to guide future standardization, EQS development, and the prioritization of sustainable treatment pathways as regulatory and analytical capacities evolve.

## 3. Physicochemical Properties and Environmental Behavior of Losartan

### 3.1. Chemical Structure and Properties

Losartan is an AT_1_ type ARB used for both cardiovascular conditions and hypertension. Losartan is chemically stable and has a hydrophobic character due to the chlorine component in its structure, a substituted biphenyl nucleus, and a tetrazole ring and imidazole heterocycle. The molecular design of Losartan has a meaningful impact on its physicochemical properties, which have a bearing on its environmental fate [16,17]. The Losartan chemical formula is C_22_H_23_ClN_4_O, with a molar mass of 422.9 g mol^−1^. Because of the presence of the tetrazole group, losartan dissociates at an approximate pKa value of 4.9 which contributes to its weak acidity. At typical environmental pH levels (approximately 6.0–8.0), losartan is almost exclusively in its anionic state and has a significant effect on its mobility in water and interaction with solids in aquatic environments [18]. Losartan’s log Kow value for the neutral form is approximately 4.0–4.3, indicating that it has moderate hydrophobicity in this form. Nevertheless, the predominant presence of losartan in its ionized state at typical environmental pH values reduces the likelihood of partitioning into organic phases and biological tissues resulting in a lower log D value compared to log K_ow_.

Losartan’s solubility is considered to have moderate to high solubility, with the amount of solubility being largely dependent on how acidic/basic and how the chemical was made. The pharmaceutical form of losartan that is most frequently utilized, losartan potassium, is the most soluble (a maximum of 100 mg L^−1^). As a result, losartan is more likely to remain in an aqueous state after being removed from the body and entering the sewer system [19]. Another parameter to consider when predicting how losartan will behave in the environment is the organic carbon–water partition coefficient (log K_oc_). Studies have determined that losartan’s log K_oc_ should fall between 2.5 and 3.1, meaning that it should have moderate mobility in soil and sediment. The physicochemical properties of losartan, because it is ionizable around neutral pH, has high solubility, and is also chemically stable, are additional reasons why losartan can be detected in surface waters and urban wastewaters and typically at concentrations between ng L^−1^ and µg L^−1^ [20]. Table 1 provides a summary of the major characteristics of losartan.

### 3.2. Environmental Fate and Persistence in Natural Systems

The liver extensively metabolizes losartan after oral administration, primarily via cytochrome P450 enzymes CYP2C9 and CYP3A4. The metabolism of losartan produces an active metabolite (EXP-3174) that possesses greater pharmacological activity than losartan itself, with both losartan and its metabolites excreted, primarily via urine or feces, to municipal wastewater treatment facilities [21]. Because the ionized form of the molecule predominates at ambient pH, losartan sorption in soils and sediments is typically moderate to low. Anionic chemicals are less likely to be retained in solid particles because they typically have a weaker attraction for hydrophobic organic materials [16]. However, electrostatic interactions and surface complexation processes can take place in soils that are rich in clays, metal oxides, or organic matter, which can increase the compound’s adsorption to some extent. However, in most cases, these processes are insufficient to stop its environmental migration [22].

Losartan is highly mobile in aquatic environments because of its high solubility and minimal retention in solid particles. As reported by the authors of this study and corroborated by many environmental studies, Losartan is detected in various aquatic environments (rivers, lakes, and in effluent from sewage treatment plants) successfully over a wide range of countries globally [20]. Moreover, traditional wastewater treatment facilities utilizing activated sludge technology lack effective losartan removal capabilities. Environmental studies have shown variability in target removal efficiency, ranging from 20% to 70% depending on hydraulic residence time and other parameters, such as operational conditions [23]. Despite having a very high log K_ow_ value when neutral, Losartan is classified as having a low to moderate potential for bioaccumulation.

Because of the high percentage of ionized species in the natural environment, the majority of pharmaceutical compounds have little or no potential for bioaccumulation due to their BCF being less than 100 [24]. Nevertheless, long-term exposures to very low concentrations of pharmaceuticals in the environment can result in adverse sublethal effects on aquatic organisms, such as physiological changes and disruptions in normal functioning of the endocrine system [25,26]. The presence of active metabolites and transformation products resulting from environmental changes can significantly influence the persistence of losartan. The primary metabolite of losartan, EXP-3174, has also been detected in surface water and wastewater and is much more potent than losartan at blocking AT receptors. The potential for additional transformation products produced during photodegradation or biotransformation to harm the environment or its organisms adds to the uncertainty in evaluating the risks associated with pharmaceutical products.

**Figure 1:**
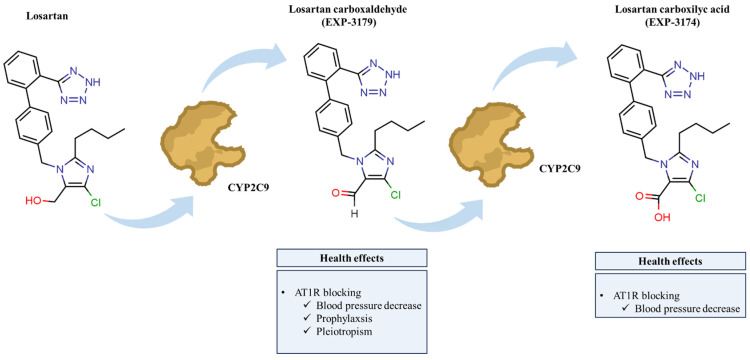
Losartan metabolic path via CYP2C9 and metabolite formation of EXP-3179 and EXP-3174.

The metabolite EXP-3174, which is 10–40 times more potent as an AT_1_ receptor antagonist than losartan and mediates the majority of its angiotensin type 1 receptor (AT1-R) blocking actions, is produced from around 14% of an oral dosage of losartan. Although losartan was thought to have no agonist qualities, EXP3179, a significant intermediate aldehyde metabolite, has been found (Figure 1) [27]. Although EXP3179 lacks AT_1_ receptor blocking function, it has strong anti-inflammatory effects by suppressing endothelial cyclooxygenase (COX)-2 expression. Additionally, it prevented the production of COX-dependent thromboxane A2 and prostaglandin F2α in vitro, as well as the overexpression of intercellular adhesion molecule (ICAM)-1 mRNA. Additionally, in cultured endothelial cells, EXP3179 triggered the activation of endothelial nitric oxide synthase via a PI3-kinase/Akt pathway downstream of VEGF receptor 2 [28]. In a study in *Hypertension*, Kappert et al. examined the hypothesis that EXP3179-mediated PPAR-γ activation implies a novel mechanism for the anti-inflammatory and antidiabetic actions of losartan because ligand-activated PPAR-γ also exerts anti-inflammatory actions (inhibiting the action of pro-inflammatory transcription factors like AP-1 and nuclear factor kB) and represses COX-2 promoter activity and mRNA expression (interacting with the c-jun component of the AP-1 complex) [29].

Losartan’s environmental durability is a product of both structural stability and comparatively slow rates of natural degradation in both terrestrial and aquatic environments. One of the primary natural processes of losartan alteration in surface waters exposed to sun radiation is photodegradation. UV radiation absorption can cause photochemical processes that produce structurally unique oxidative intermediates and transformation products [23]. Under normal environmental conditions, chemical hydrolysis is not thought to be a dominant degradation process for losartan. The slow rate of the process is a result of the stability of the aromatic ring structure and the absence of many functional groups that are very susceptible to hydrolysis. The environmental characteristics of losartan and other ARBs are compared in Table 2 [30,31]. Losartan is less hydrophilic than other ARBs (such as irbesartan) but more lipophilic than telmisartan, indicating that losartan has average hydrophobicity compared to the other ARBs. Due to its stable aromatic structure, losartan has moderate persistence in aquatic environments; however, due to its ability to become ionized, it remains in the aqueous phase for a prolonged period of time and can easily migrate within aquatic environments. Because of its high-water solubility, potassium losartan can readily diffuse and disperse throughout wastewater effluent (the most common way that losartan is found in urban effluent). However, other ARBs (specifically, telmisartan) that are more hydrophobic are generally more likely to sorb to sediments and bioaccumulate within aquatic organisms, which may cause other forms of ecotoxicological risk [32].

## 4. Environmental Occurrence and Ecological Risk

### 4.1. Environmental Occurrence and Distribution in Aquatic Matrices

Losartan can be found in almost all environmental matrices at high levels as a result of the continued worldwide use of this compound. The prevalence of Losartan within our waterways is a result of the sheer amount of it that is consumed, its excretion from human/animal bodies in unaltered form, and the failure of wastewater treatment plants to successfully remove pharmaceutical micropollutants from water. After taking Losartan orally, most of it and all of its metabolites will be excreted from the body via feces and urine, eventually traveling to the wastewater treatment plants, and therefore entering the waterway like all other substances that could be carried via untreated sewage [33]. Estimates indicate that Losartan concentrations in effluent released from wastewater treatment plants generally range from several hundred ng L^−1^ to several µg L^−1^. According to previous studies of wastewater treatment plant effluents, the concentrations upon treatment are approximately 1–2 µg L^−1^. However, studies of hospitals and industrial discharges may have concentrations significantly greater than those values, some as high as several µg L^−1^ or even into the mg L^−1^ range in extreme scenarios in which pharmaceuticals are manufactured [5].

Higher concentrations of losartan are present in densely populated or poorly treated sewer systems, but are typically lower in surface waters, with most concentrations ranging from 0.17 to 36.5 ng L^−1^ [5]. The drug has also been discovered within coastal regions of Brazil, with 100% of seawater samples from Santos Bay containing concentrations of losartan from 0.20 to 8.60 ng L^−1^ demonstrating ongoing discharges into the marine environment from urban sewage [25]. Concentrations found in coastal rivers in São Paulo ranged from 0.10 to 8.42 ng L^−1^, indicating the wide distribution of pharmaceutical residues across aquatic environments [34]. Data are limited, but losartan has also been detected in groundwater and sewage sludge, especially in areas with biosolids application to crops or infiltration of treated effluent. The detection of losartan in multiple environmental media shows the ubiquitous nature of pharmaceutical contaminants due to continuous human consumption and uncontrolled discharge of inadequately treated wastewater.

Table 3 provides a summary of the environmental prevalence of losartan in various aquatic environments, demonstrating significant trends related to the sources of contamination, mechanisms of transport, and the potential for ecological exposure. Losartan generally appears in the greatest concentrations in the effluents of wastewater treatment facilities. These findings indicate that the primary source of losartan emissions is from domestic sources, as this drug is commonly prescribed for hypertension and is excreted in either unchanged form or as active metabolites. Additionally, the presence of losartan in treated effluents, combined with the fact that traditional biological treatment systems (e.g., activated sludge) are not fully effective at removing this type of pharmaceutical micropollutant, indicates that losartan is continuously introduced into downstream water bodies [35]. In natural aquatic environments, particularly rivers and surface waters, losartan concentrations are orders of magnitude lower and typically less than 1 ng L^−1^. The predominant reasons for this decline include dilution throughout the aquatic environment, photochemical degradation, and hydrological dilution processes. Nonetheless, the persisted presence of the compound in these concentrations indicates that contributions from the urban effluents are persistent and sustained enough to provide consistent detectable concentrations over time. This has also been shown by studies of pharmaceuticals located globally that showed prevalence of antihypertensive drugs within the more frequently detected compounds within urbanized rivers/streams impacted by urbanization.

Additionally, the existence of losartan in marine and coastal environments (as observed in Brazilian coast research) is another aspect of the information given above. In the case of marine waters (aquatic systems), the chemical was detected at very low concentrations (0.2–8.7 ng L^−1^), which shows that this type of habitat can be the last recipient of this class of pharmaceutical pollutant after they enter coastal systems from metropolitan watersheds. It is especially concerning because the oceanic environment allows the dispersal of this class of contaminants, increasing the extent of ecological exposure to aquatic ecosystems, facilitating the bioaccumulation of contaminants, and causing potential sublethal toxicity to aquatic organisms. The data presented in this table suggest that there is a general order of environmental gradients for pharmaceutical contaminants where concentrations are highest closest to their source of emission (effluents from WWTPs) and decrease with increasing distance from the effluent sources due to dilution and degradation as they move through aquatic systems. However, the widespread occurrence of losartan in multiple environmental matrices may indicate significant persistence such that it may be moved through hydrological systems and play a role in diffusive contamination of aquatic ecosystems.

### 4.2. Analytical Methods and Detection Techniques

Losartan is challenging to analyze in environmental matrices due to the complexity of the substances, the low anticipated quantity of material, and the selectivity criteria that make it difficult to differentiate from structurally identical interferences. A number of critical validation criteria pertaining to methodology as well as analysis of the limitations inherent within each method will be covered in this chapter, ranging from sample preparation to instrumental detection methods employed in various literature sources. In order to reach ecotoxicological significance, pre-concentration is needed to achieve detection limits, as losartan is present in very low levels in environmental water samples. The most common sample preparation method for pharma analysis within aqueous matrices is solid phase extraction; cartridges utilizing reverse phase (C18, C8) or mixed ion exchange (e.g., Oasis HLB, Strata-X) sorbent phases are typically used for the extraction of amphoteric pharmaceuticals like losartan due to their ability to extract using a variety of pH levels [48]. Generally, a sample pH level of around 3 is achieved to increase analyte recoveries by regulating the ionization state of the analyte or optimally extracting analytes from solution. SPE offers advantages in terms of high pre-concentration efficiency (factors of 100 to 1000×), consistent reproducibility, and compatibility with automation; however, it also poses disadvantages, such as the high cost of disposable cartridges, potential for column clogging in high solid content matrices, and the need to optimize the method for each unique matrix [49]. The dSPE and solid phase micro-extraction have gained popularity as environmentally friendly alternatives to solvent-based sample preparation methods due to their lower use of organic solvents and capability to utilize less material for preparation. However, the use of these two methodologies is becoming less common for the analysis of losartan in complex sample matrices due to issues with extraction phase depletion and competition for adsorption sites in sample matrices that contain high levels of dissolved organic matter. Although liquid–liquid extraction has been an important historical practice that has ultimately lost much ground to SPE in environmental field studies due to greater solvent use, lower selectivity and difficulty with automation, after a primary first extraction step, both methodologies are appropriate for nonaqueous matrices (e.g., sediment, biological tissues) [50].

The analysis of polar and heat-sensitive pharmaceuticals in the environment typically uses the latest and greatest technology available, which consists of the combined use of similar technologies for both the separation and detection of these types of compounds from the environment, i.e., mass spectrometry (MS) and high-performance liquid chromatography (HPLC) or ultra-high-performance liquid chromatography (UHPLC), respectively. For particularly low concentrations of substances (usually <5 ng L^−1^), the use of tandem mass spectrometry QqQ in MRM, when compared to HPLC and UHPLC, provides the ability to make precise and accurate determinations regarding the pharmacological/physicochemical attributes of the substances being tested by analyzing the specific precursor–product transitions [51]. Non-target detection and identification of unknown metabolites and transformation products are made possible by LC-HRMS, which corresponds to high-resolution and precise mass spectrometers [52]. Their extensive screening capabilities and retrospective data reprocessing make them invaluable for research identifying and characterizing environmental transformation pathways, even though they occasionally show somewhat lower sensitivity than QqQ [53]. HPLC with UV/Fluorescence Detection are techniques based on traditional detectors that are affordable and appropriate for large concentrations or less complex matrices (such as industrial effluents) [54]. However, they show reduced selectivity in matrices with co-eluting interferents, and their detection limits (usually >1 µg L^−1^) are unsuitable with the majority of surface and drinking water scenarios [55]. The validation of the procedure in accordance with international norms is the only way to ensure the trustworthiness of environmental occurrence data. The primary quality control parameters are summarized in Table 4 [48].

Losartan presents a number of unique analytical challenges. For instance, losartan can degrade hydrolytically or photochemically during collection, transportation, and storage, which can lead to instability and ex situ transformation [56]. To maintain sample integrity, it is advised to apply preservatives, store the sample in a refrigerator at 4 °C, and analyze it within 48 to 72 h. Additionally, structurally identical metabolites may cause interference. In order to prevent overestimating concentrations, the active metabolite EXP-3174 and other derivatives must have sufficient chromatographic resolution and particular MRM transitions because they share physicochemical properties with the parent substance [51]. Research indicates that losartan can biodegrade into valsartanic acid, a highly persistent transformation product that needs close observation [15]. Another challenge to be taken into account is matrix effects in saline fluids and sediments. The presence of hydrophobic interferents and the suppression of ionization by salts make the analysis of seawater or sediment extracts more difficult [57]. It may be essential to employ techniques such as sample dilution, specialized SPE phases, or extra silica gel cleanup.

Future viewpoints and certain new trends should be taken into consideration in order to overcome these challenges. Automation and high-throughput screening, where the combination of UHPLC-MS/MS and online sample preparation technologies enables quick analysis of huge sample series, lower human error and boost lab output [58]. Although these techniques still need strong validation for regular environmental application, the development of electrochemical or optical biosensors for in situ losartan detection provides a promising frontier for real-time monitoring. Lastly, the identification of unknown transformation products is made easier by the combination of LC-HRMS with spectrum libraries and in silico methods (such as fragmentation prediction), which expands our knowledge of losartan’s environmental fate [59]. It is advised to validate the method for various water types (surface, groundwater, drinking water, marine, and, if applicable, sediments or biota); participate in interlaboratory programs to confirm the accuracy and traceability of results; and, when appropriate, include detailed LOD, LOQ, recoveries, matrix effect, and expanded uncertainties in publications to strengthen the comparability of data between studies and regions.

### 4.3. Ecological Risk Assessment and Ecotoxicity

The PEC and the PNEC are typically compared in order to determine the environmental risk of developing pollutants. The Risk Quotient (RQ) is calculated as the ratio of the Predicted Environmental Concentration (PEC) to the Predicted No-Effect Concentration (PNEC). Aquatic organisms are considered at low risk if the RQ value is less than 0.1, at moderate risk if it is between 0.1 and 1, and at high danger if it is greater than 1. Losartan may provide a low to moderate ecological danger in aquatic habitats, according to recent studies, depending on local concentrations and the species’ susceptibility [34]. This medication’s ecotoxicity has been studied at various trophic levels. Fish, crustaceans, and microalgae all react to the chemical in different ways, according to experimental testing. The microalga *Desmodesmus subspicatus* exhibited the lowest EC_50_ (72 h ≈ 27.9 mg L^−1^) among the tested species, indicating relatively higher sensitivity compared to the fish Astyanax altiparanae and the microcrustacean *Daphnia magna*, although this value corresponds to low acute toxicity according to standard ecotoxicological classification criteria (GHS: EC_50_ > 10 mg L^−1^) [60]. Chronic exposure to low concentrations and the simultaneous presence of many medicines can have significant sublethal effects, even if these values are well above usual environmental concentrations. Additionally, research on marine species has shown genotoxic and biochemical changes following exposure to environmentally relevant amounts, such as DNA damage in mollusks exposed to the substance and the activation of biotransformation enzymes [61].

The acute toxicities of losartan on various aquatic species are low; however, mounting evidence indicates the potential for complex and negative long-term ecological effects as a result of its continued presence in the environment. Certain cardiovascular medications are known to affect hormonal pathways found in both vertebrate and invertebrate species and are therefore one of the most discussed possibilities for losartan causing endocrine disruption in aquatic species. Long-term exposure of aquatic species to losartan will likely alter osmoregulatory and cardiovascular-related physiological processes; however, the specific pathways of action underlying these changes have not yet been elucidated [5].

Chronic toxicity is an additional reason for concern regarding sub-lethal effects such as genotoxicity, oxidative stress, and enzymatic changes. For example, it has been shown that ng L^−1^ concentrations on the order of hundreds are capable of altering physiological and metabolic indicators of marine organisms, which suggests that prolonged periods of exposure could impact biological processes associated with growth shaped by reproduction and development [62]. Furthermore, there has been some recent information in the literature that relates to microbial resistance and its potential development due to the presence of pharmaceutical residues in the aquatic environment. Although antibiotics are the most frequently correlated to this effect, some studies have indicated that various forms of pharmaceutical compounds may also indirectly provide selective pressure on microbial communities causing shifts in the microbiological community composition and promoting the selection of organisms with characteristics that have a greater tolerance for xenobiotic substances [5].

## 5. Conventional Removal Technologies

Traditional wastewater treatment technology has seen a major shift in its effectiveness due to the presence of pharmaceuticals in the municipal wastewater stream. Originally designed to remove nutrients and biodegradable organic matter, traditional biological systems were not designed to handle the very stable manufactured organic chemicals that exist in many pharmaceuticals. The result is that many of these pollutants can pass through treatment systems and enter receiving aquatic ecosystems. Losartan, one of the hypertension medications commonly found in urban effluents, has a prolonged environmental behavior and is only partially eliminated by traditional biological treatment [63,64]. According to recent research, a number of variables, such as the compound’s molecular structure, the system’s operating conditions, the solids’ retention period, and the makeup of the microbial communities, affect how effectively pharmaceutical micropollutants are removed in wastewater treatment facilities. According to Wade and Olawade [65], compounds such as losartan and other complex aromatic compounds often have poor biodegradability and thus are poorly removed by conventional treatment methods; in addition, their incomplete removal can lead to a continuous discharge of these compounds into natural aquatic ecosystems. Recent studies demonstrate that wastewater treatment facilities act as both a diffuse source and partial barrier of micropollutants [66].

### 5.1. Removal by Conventional Biological Processes

All around the world, wastewater treatment plants utilize the activated sludge process to treat wastewater. During treatment, heterotrophic bacteria in the AS process utilize aerobic metabolic processes to metabolize dissolved organic matter. However, there are two main mechanisms through which pharma chemicals are typically removed: sorption to sludge biomass and partial microbial biodegradation [67]. The extent of each mechanism can vary and is particularly dependent upon the respective hydrophobicity and affinity of the compound for sludge organic matter. In the case of losartan, research indicates that sludge sorption is likely to be a significant method of elimination; however, there may be limited levels of biodegradation. Recent studies show that pharmaceutical removal rates from CAS systems vary considerably [68]. Depending on the circumstances of the treatment plant, removal rates of antihypertensives and other aromatic micropollutants are generally between 20% and 60% [68]. Several factors are responsible for the variability of these removal values including sludge age, temperature of the system, amount of organic load in the influent, and adaptability of the microorganisms that break down pharmaceuticals in the CAS [69]. Additionally, as the microbial community preferentially utilizes the more energetically favorable carbon sources, the presence of more easily biodegradable organic substrates will result in a reduction in the degradation of pharmaceutical compounds [69].

Membrane bioreactors (MBRs) are a more technically advanced system for performing the same functions as traditional activated sludge systems. With MBRs, biological treatment and separation using microfiltration or ultrafiltration membranes can take place at much greater concentrations of biomass. As a result, this will allow for longer interactions between micropollutants and biomass, an increased SRT, and a greater diversity of microbes within MBR systems [70], all of which may lead to enhanced degradation of recalcitrant organic pollutants. According to recent research, MBR systems can outperform CAS systems in terms of removal efficiency, especially for chemicals that are only mildly biodegradable. Nevertheless, it is rarely possible to completely eliminate structurally stable medications, even in these sophisticated systems. Additionally, the contamination may be transferred to the biological sludge rather being fully mineralized due to the retention that the membranes encourage. The elimination efficiency of biological technology is shown in Table 5 [71].

Pharmaceutical micropollutant removal in wastewater treatment plants is highly variable, according to recent global data compilations. Depending on the method used and the compound’s chemical composition, typical removal efficiency for various pharmacological classes ranges from 30% to 80% in thorough literature evaluations [66]. Nevertheless, expected removals remain modest for some highly persistent compounds; therefore, it is critical to find additional treatment options. Kinetic limits are another important consideration. Traditional biological processes are commonly utilized; however, their ability to effectively remove pharmaceutical micropollutants is typically minimal. These restrictions in the case of losartan are mostly related to competing with more readily degradable organic substrates, slow biodegradation kinetics, and conventional bacteria’s poor metabolic affinity [73]. The low ambient concentration of these substances, which might not be enough to trigger the development of particular metabolic pathways required for their microbial breakdown, is another crucial aspect. Additionally, new research shows that partial medication degradation during biological treatment can produce potentially permanent transformation products, the environmental toxicity of which is still unknown [74]. Given these constraints, a number of authors stress that the use of sophisticated tertiary technologies, such as ozonation, adsorption on activated carbon, and advanced oxidation processes, is frequently required to achieve higher levels of removal, and that conventional biological processes should only be viewed as an initial barrier in the control of pharmaceutical micropollutants.

While aerobic biological processes dominate the literature on losartan removal, anaerobic treatment pathways remain largely unexplored. This gap is primarily attributed to losartan’s recalcitrant aromatic structure and its anionic speciation at neutral pH, which limit biodegradability under reducing conditions. Recent studies indicate negligible removal efficiencies (<20%) in anaerobic digesters, likely due to the absence of oxygen-dependent enzymes required for initial ring hydroxylation and cleavage. Consequently, current research and full-scale applications predominantly focus on aerobic activated sludge and membrane bioreactor systems, where higher microbial diversity and oxidative metabolic pathways yield more consistent, albeit partial, removal rates.

### 5.2. Physicochemical Treatment Processes

#### 5.2.1. Coagulation and Flocculation

In water treatment facilities, coagulation/flocculation is a standard procedure that involves adding organic polymers or metallic coagulants (such as ferric chloride or aluminum sulfate) to destabilize colloidal particles and dissolved organic debris. The effectiveness of removing medications such as losartan from the emerging contaminants is limited by the primary interactions occurring between losartan and hydrolyzed coagulants. Losartan (which has two ionizable functional groups) is able to react with hydrolyzed coagulants in three different manners: via (i) charge neutralization with ions from the coagulant, (ii) sweep flocculation of coagulants and losartan into the precipitated metal hydroxide flocs, or (iii) surface complexation with active sites on the flocs. However, due to the dissolved non-ionized molecule form of losartan typically remaining stable in the aqueous phase, removal of losartan via this method is limited. Overall, studies demonstrate that conventional coagulation will remove losartan at moderate rates (generally 10–40% removal), depending on the method and conditions used (e.g., coagulant type, dosage, pH, alkalinity). Additionally, traditional methods of treatment (e.g., flocculation or coagulation) for the emerging contaminants will often fail due to their low molecular weights, hydrophilicity, and chemical stability [5].

There are several operational considerations for operational efficiency. For instance, pH directly affects adsorption/complexation affinity by regulating the ionization state of losartan and the speciation of metallic coagulants. Careful estimation of the coagulant dose is necessary since high doses may result in increased residual turbidity or particle restabilization without a commensurate increase in drug removal [75]. Additionally, the kind of aqueous matrix should be considered, as competing ions and NOM may limit the coagulant’s active sites for interaction with losartan [76]. Isolated coagulation/flocculation is not regarded as a reliable method for removing losartan on a large scale. However, it can lower the organic load and shield units that are more susceptible to fouling when used as a pretreatment for later procedures like adsorption and membranes.

Preliminary experiments were carried out in a study to look at the sorption potential of various organic micropollutants in polystyrene and polyethylene and to assess how well these microplastics were removed during coagulation trials using iron and manganese coagulants [77]. Eight synthetic chemical substances from three distinct categories, pharmaceuticals, personal care items, and endocrine disruptors, were used in the sorption assays. When polystyrene was employed as a sorbent material, there was a notable removal of microplastics from the hypertension medications losartan and valsartan. These substances sorbed slowly and gradually; after 168 h, 20% of valsartan and 59% of losartan were sorbed. However, sulfamethoxazole, bisphenol A, and parabens did not exhibit sorption. Polystyrene is eliminated at a higher proportion than polyethylene, reaching 92% and 72%, respectively, according to the coagulation trials. Ferric chloride did not enhance the removal of polystyrene; instead, the addition of ferrous sulfate or magnesium sulfate produced the maximum removal. Polyethylene removal was enhanced by magnesium sulfate. The factors influencing the sorption of losartan and valsartan on microplastics and the processes controlling the elimination of polystyrene and polyethylene during coagulation should be further studied [77].

#### 5.2.2. Adsorption on Activated Carbon

One of the best methods for eliminating polar organic pollutants from water, including medications like losartan, is adsorption on AC. Van der Waals forces, hydrophobic contacts, hydrogen bonds, and, occasionally, electrostatic interactions mediate the physicochemical interaction between the analyte and the porous surface of the adsorbent [78,79,80]. Losartan preferentially interacts with hydrophobic areas of the AC surface because of its amphiphilic nature and aromatic groups. π–π type interactions between the drug’s aromatic rings and the graphitic structure of the activated carbon are more common at pH values near the adsorbent’s point of zero charge (pH_PZC_). Electrostatic interactions can enhance the process at a pH that is far from the pH_PZC_, particularly if the adsorbent and analyte have complementary charges [81]. Critical factors will determine how efficient the procedure is. The adsorption capacity and selectivity are determined by surface area, pore volume, pore size distribution, and surface chemistry (oxygenated groups) (adsorbent characteristics) [5]. The pH of the solution is another factor that affects electrostatic interactions by affecting the ionization of losartan and the surface charge of the active compound. Other sources of competition for active sites such as natural organic matter and inorganic ions, or other organic pollutants, may reduce the capacity for specific adsorption of losartan. Lastly, temperature also plays a role since exothermic adsorption processes can become less efficient as the temperature rises, though this effect is usually negligible in ambient ranges [82].

Granular activated carbon is frequently utilized in fixed-bed filters in water treatment facilities and effluent polishing stations in terms of practical application and regeneration. The increasing saturation of the active areas necessitates material replacement or thermal/chemical regeneration, which affects operating expenses [83]. Research on in situ regeneration techniques and inexpensive adsorbents (such as biochar made from rice or coconut husks) represents frontiers for the process’s economic sustainability. In a groundbreaking study, a magnetic stir bar was coated for the first time utilizing the sol–gel method with both the ionic liquid 1-ethyl-3-methylimidazolium hexafluorophosphate and nickel: zinc sulfide nanoparticles (Niz’s NPs) deposited on CA (Ni:ZnS-CA) [84]. Losartan and valsartan were then extracted sorptively using a magnetic stir bar as model compounds. High-performance liquid chromatography with UV detection was used to quantify the isolated analytes. By adjusting the factors influencing sorptive extraction on a magnetic stir bar, such as sample solution pH, ionic strength, extraction time, desorption solvent volume, desorption time, and stirring speed, the best extraction performance for losartan and valsartan was achieved. The most crucial parameters were determined using fractional factorial design, and they were subsequently optimized using central composite design and the desirability function. Wide linear ranges of 0.4–50 μg L^−1^ and 0.5–50 μg L^−1^ and good relative standard deviations (at the 5 μg L^−1^ level and n = 6) of 4.4% and 4.9% were found for losartan and valsartan, respectively, under ideal experimental circumstances. The developed method’s detection limits (S/N = 3) for losartan and valsartan were 0.12 μg L^−1^ and 0.15 μg L^−1^, respectively, with enrichment factors of 188.6 and 184.8 times. Losartan and valsartan levels in urine and plasma matrices were successfully determined using the described approach [84].

#### 5.2.3. Membrane Processes

**Figure 2:**
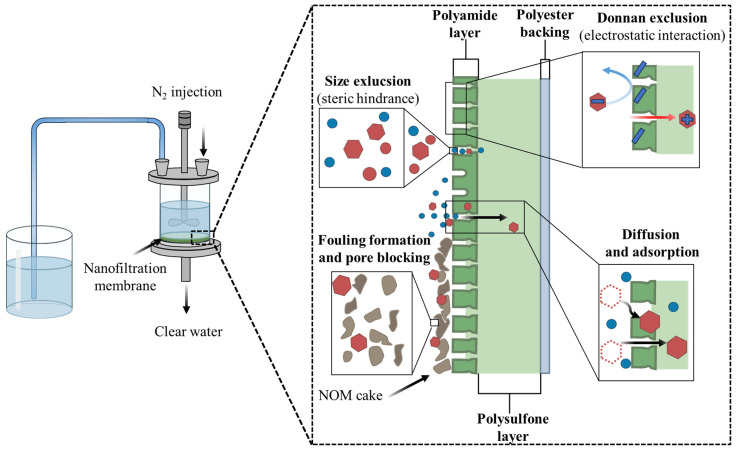
Experimental setup and losartan mechanistical illustration.

Membrane separation technologies (MF, UF, NF, and RO) use size exclusion mechanisms, electrostatic repulsion, and affinity interactions to provide effective physical and chemical barriers against emerging contaminants. The best configuration of membranes for losartan (Mw ≈ 422.5 g mol^−1^; dimensions of approximately 1.2 nm) are NF and RO membranes because they have a surface charge that allows electrostatic repulsion of ionic species, and have pore sizes in the 0.5–2.0 nm range. Because the bulk of the medication is in solution and not associated with colloidal particles, retention via MF/UF is typically negligible (<<30%). Based on results of experiments conducted, NF and RO membranes are capable of removing over 95% of losartan from synthetic fluids and secondary effluent; the rejection is dependent on the ionic strength, trans-membrane pressure, recovery and pH of the feed [85,86]. Several studies have been conducted using NF membranes to reject losartan from sewage and hospital wastewater [87]. In these studies, it was found that the NFX commercial nanofiltration membranes had consistently good NF membrane reject rates greater than 80%, whereas other NF membranes of varying designs demonstrated reject rates for losartan ranging from 10 to 90% [87]. A flow filtration cell was used in this research to evaluate the performance of the NF membrane, as illustrated in Figure 2. This DEF cell (DEF cell, Sterlitech, HP4750, Auburn, WA, USA) was equipped with a stirrer that had a capacity of 300 mL and was connected to a nitrogen gas source (DEF cell-0007; nitrogen gas; pressure). Each experimental run was completed with 200 mL of sample, while the working area of the DEF cell membrane was 14.6 cm^2^; the permeate flux was recorded every five minutes using a graduated cylinder. The fouling behavior of the membranes was observed and recorded at three different membrane pressures of 10, 20, and 30 bar [87].

One of the problems associated with the membrane process is fouling, which refers to the build-up of organic waste, colloids, or biofilms on the membrane. The build-up of this debris reduces the amount of permeate produced and can adversely affect the way the membrane separates materials. To ensure ongoing use (sustainability) of this membrane process, pretreatment techniques along with routine chemical cleaning are necessary [88]. Another challenge is the disposal of tailings, which are produced during membrane processes. Tailings are the concentrated solution that is produced during the separation process and contains all of the impurities that have collected on the membrane. There is an economic and environmental challenge related to the proper disposal of tailings. The amount of pressure (between 10 and 150 bars) required for RO indicates how much energy may be needed to use RO technology, which limits its use in regions that do not have ready access to much energy. Researchers hope to develop hybrid membranes that combine high permeability with increased selectivity and resistance to fouling (by including nanoparticles, or functionalized polymers), thereby improving membrane performance [89]. The use of hybrid membrane configurations that incorporate AOPs, such as catalytic membranes, may be an innovative way to remove and degrade pharmaceutical compounds simultaneously [90]. Table 6 is a comparison of the physicochemical processes reviewed; it describes the major types of mechanisms utilized, ranges of efficiencies for losartan, and possible operational advantages and disadvantages of each type of process.

When selecting as well as scaling up physicochemical processes for removing losartan, consideration should also be given to other factors such as economic feasibility, environment-friendly operation, as well as how well the chosen technology integrates into any existing treatment processes. Multi-barrier technologies are very useful for limiting the risks of newly emerging contaminants in aqueous resources, e.g., utilizing membranes in conjunction with the adsorptive capacity of activated carbon for polishing purposes, and coagulating prior to treatment.

## 6. Advanced Remediation Technologies

Losartan’s presence in aquatic environments represents an important challenge for conventional wastewater treatment plants since the complex chemical structure of losartan (which includes both imidazole and tetrazole rings) combined with its pseudo-persistent nature (meaning that it will not be easily biodegradable) allows its significant passage through the secondary biological treatment processes used at these plants. As a result, there should be additional tertiary polishing technologies used to remove losartan from wastewater in addition to the standard secondary biological practices already in place today. Additionally, AOPs appear to be a new and promising alternative method that can potentially degrade losartan into harmless by-products [93].

### 6.1. Advanced Oxidation Processes

As the fundamental mechanisms of AOPs have been extensively reviewed in recent literature, this section focuses specifically on their application to losartan degradation, emphasizing kinetic performance, operational trade-offs, and the critical challenge of transformation product (TP) toxicity. Detailed comparative parameters, energy requirements, and process limitations are consolidated in Table 7 to avoid redundancy and direct readers to a structured, losartan-specific evaluation. Thus far, the most well-studied advanced oxidation process for eliminating pharmaceuticals from water is ozonation. Ozone reacts with Losartan through both direct and indirect oxidation mechanisms; direct oxidation occurs at electrically rich sites (e.g., imidazole ring), whereas indirect oxidation creates hydroxyl radicals, which subsequently acts to oxidize losartan at alkaline PHs. Studies have shown that Losartan can be completely destroyed ~90% of the time within 10 min at the appropriate conditions. The performance of a matrix affects how effective it will be; if there is ample dissolved organic material that attracted free radicals from the ozone, it would considerably reduce the effectiveness [94]. A significant criticism of ozonation relates to the formation of oxidized byproducts; for example, if bromide is present in the water and ozonated, bromates (a known carcinogen) may form. Additionally, other contaminants can form via ozonation (aldehydes), some of which may be as harmful as or even more so than the parent compound. Ozone’s limited effectiveness due to low water solubility and high cost of generating or utilizing ozone on-site also inhibit its use without careful consideration of dosage optimization [95].

The photolysis of hydrogen peroxide under ultraviolet light, which produces OH radicals, is the basis of the UV/H_2_O_2_ process. Because losartan has a low molar absorptivity in the UV-C regions, this method is more effective than direct photolysis (UV only). Accelerated deterioration is made possible by the oxidant and radiation working together. However, the high energy consumption of UV lamps and the requirement for exact stoichiometric dosing of H_2_O_2_ sometimes undermine the viability of the UV/H_2_O_2_ process. The effectiveness of the process can be decreased by excess peroxide acting as a scavenger of OH radicals (OH + H_2_O_2_ → HO_2_ + H_2_O). Additionally, the wastewater’s turbidity and color can prevent radiation from penetrating, necessitating physicochemical treatments [96]. In one investigation, losartan potassium’s genotoxicity and acute and chronic ecotoxicity were assessed both before and after UVC/photolysis and UV/H_2_O_2_ processes [33]. After 30 min, both methods’ effective losartan degradation rates exceeded 99.99%. Additionally, unidentified byproducts were seen to occur during deterioration throughout the treatments. After 480 min, both procedures decreased both acute and long-term toxicity. Nevertheless, after 240 min in the UV/H_2_O_2_ process, *Daphnia magna* and *Daphnia subspicatus* demonstrated sensitivity to the byproducts [33]. Losartan produced genotoxicity in Daphnia magna cells both before and after 480 min of UV/H_2_O_2_ exposure. This highlights the significance of (i) employing species from various trophic levels; (ii) monitoring AOPs bioanalytically; and (iii) examining sublethal toxic effects utilizing biomarkers like genotoxicity. The 480 min UVC/photolysis therapy is the most appropriate for losartan elimination under the circumstances utilized in this investigation, taking into account the results of acute and chronic ecotoxicity in addition to genotoxicity [33].

Iron is used as a catalyst in the Fenton (Fe^2+^ + H_2_O_2_) and photo-Fenton (addition of UV/Vis radiation) processes to break down peroxide. In these systems, losartan is efficiently broken down, with photo-Fenton showing higher reaction rates because of ongoing Fe^2+^ regeneration and extra ferric complex photolysis [97]. The primary significant drawback of the traditional Fenton process is the operating pH constraint (ideally between 2.5 and 3), which necessitates acidification of the wastewater and subsequent neutralization, producing substantial amounts of ferrous sludge that needs to be properly disposed of. Although solar photo-Fenton reduces energy expenditures to some extent, continuous flow application still faces a technological challenge in recovering the homogenous catalyst. Although heterogeneous catalysts exhibit lower catalytic activity than homogeneous systems, recent research focuses on them to reduce sludge production [98]. A study used the photo-Fenton method with UV-Vis LED to optimize the mineralization of losartan and hydrochlorothiazide [99]. Fe^2+^ at 10 mg L^−1^ and H_2_O_2_ at 100 mg L^−1^ were the optimal conditions that maximized mineralization efficiency after the experimental design was optimized using a Doehlert matrix and a global desirability function. Losartan and hydrochlorothiazide had high mineralization rates; after 90 min, COD elimination for both medications was about 75%. According to the kinetic model, during the first few minutes of mineralization, there were two regimes: rapid progression and slower activity. Using the UV-Vis LED-assisted photo-Fenton process, the estimated energy consumption for the mineralization of losartan and hydrochlorothiazide at a concentration of 20 mg L^−1^ in 60 min was 130 kWh m^−3^.

The desirability function is utilized as an effective method of establishing the best experimental conditions to treat wastewater of diverse characteristics. In the photo-Fenton method, UV-Vis LEDs have proven to be a viable light source [99]. The activation of persulfate (S_2_O_8_^2−^) represents a new alternative method to generate sulfate radical (SO_4_^−^) via AOPs that are more selective and provide a greater half-life than OH^−^ (E_0_ = 2.5–3.1 V). Transition metals, UV light, or heat can all be used for activation. Compared to Fenton, the activated persulfate method exhibits resilience for losartan over a larger pH range. The SO_4_^−^ nature radical is very useful in the breakdown of certain functional groups of losartan because it facilitates electron transport processes. Persulfate is more expensive than hydrogen peroxide, and adding sulfate ions to the treated effluent can lead to infrastructure deterioration and water salinization. Despite its efficiency, heat activation creates a substantial energy barrier [100].

**Figure 3:**
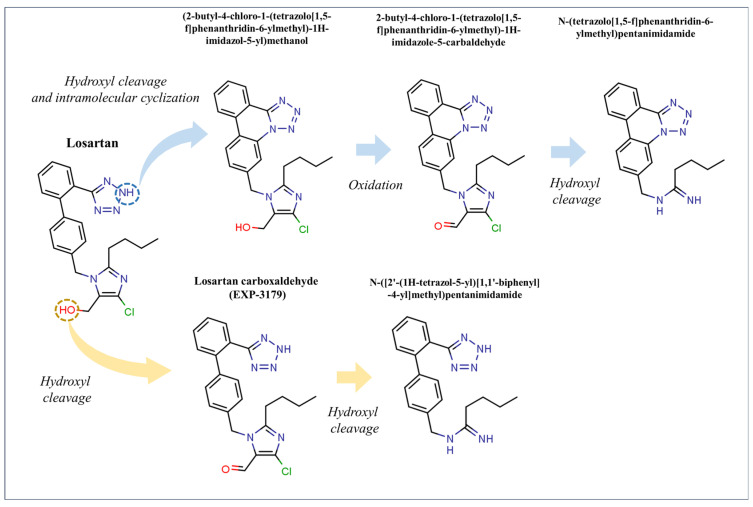
Transformation products of losartan identified during heat-activated persulfate treatment with and without ultrasound assistance, showing degradation pathways and structural modifications.

A study examined the kinetics, transformation products, and synergistic effects of removing losartan from environmental aquatic matrices using heat-activated persulfate [83]. The efficiency of losartan degradation, which follows pseudo-first-order kinetics, rises with temperature, persulfate content, and acidic conditions. The authors determined an apparent activation energy of 112.70 kJ mol^−1^ based on the corresponding apparent rate constants in the 40–60 °C range. The •OH contributes to the degradation of losartan, according to radical scavenging assays. In genuine aqueous matrices, such as bottled water and secondary sewage effluent, degradation was inhibited; nevertheless, other investigations showed that humic acid and bicarbonates had an adverse effect on its oxidation. On the other hand, the elimination of losartan was positively impacted by the addition of 250 mg L^−1^ of chloride ions. Low-frequency ultrasound and heat-activated PS demonstrated a synergistic effect; the S ratio was 1.52 in secondary sewage effluent and 2.29 in bottled water. Using HRMS and suspect and non-target screening techniques, five transformation products of losartan were found; two of these are reported for the first time (Figure 3). The majority of the discovered transformation products were shown to be more hazardous than losartan against *Daphnia magna* using the internal risk assessment algorithm, ToxTrAMs [101].

A recent study examined the breakdown of the hypertension medication losartan in water by activating persulfate using biochar made from pomegranate peel [102]. Greater catalytic activity was demonstrated by biochar pyrolyzed at 850 °C, which was associated with a bigger surface area and a higher concentration of minerals on its surface. Interestingly, losartan degradation was greatest at pH 3, despite the fact that adsorption is preferred at alkaline pH. Losartan broke down according to pseudo-first-order kinetics. Persulfate greatly accelerated the reduction in losartan, although the aqueous matrix’s organic and inorganic components such as humic acid, sodium chloride, and bicarbonate inhibited oxidation. Studies using radical scavengers showed that singlet oxygen, hydroxyl radicals, and sulfate radicals all contributed to the breakdown of losartan, with the former being the predominant species. The technology demonstrated acceptable steady-state performance using a continuous flow reactor, with 90% losartan elimination. The technology demonstrated good steady-state performance using a continuous flow reactor, removing 90% of the losartan in 114 h. A slight decline in performance was then noted, which can be explained by changes to the catalyst surface and acidity-induced mineral dissolution [102].

Another study used an eco-friendly technique to modify the physicochemical characteristics of biochar and used it to activate persulfate to oxidize losartan [103]. The shape and carbon phase were changed, and the surface area was greatly enhanced from 100 to 308–428 m^2^ g^−1^. Additionally, there was a considerable impact on the point of zero charge and the percentage of minerals. The medication losartan was most degraded by the acid-treated biochar among the materials tested when persulfate was present. Remarkably, at pH 3, the charcoal functioned as an adsorbent, but, between pH 5.6 and 10, the apparent kinetic constant ratio k_oxidation_/k_adsorption_ was 3.73 ± 0.03, indicating losartan oxidation. The importance of the non-radical process (singlet oxygen) was indirectly shown by free radical scavenging tests. Nevertheless, hydroxyl and sulfate radicals also played a major role in the oxidation of losartan. Due to competition between the target chemical and the components of the aqueous matrix for the biochar’s active sites and reactive species, experiments conducted in secondary effluents showed poorer efficiency than those conducted in pure water [103].

Any remediation solution for losartan needs to be critically examined beyond the “parent compound removal” metric. TPs produced by incomplete oxidative degradation have the potential to maintain or worsen pharmacological efficacy and ecotoxicity. Losartan oxidation creates metabolites including EXP-3174 (active) and other carboxylic acids and aldehydes, according to LC-MS/MS identifications. According to ecotoxicity studies using *Vibrio fischeri* and *Daphnia magna*, acute toxicity may rise during intermediate oxidation stages (before total mineralization) as a result of the production of more polar and bioavailable TPs. Therefore, rather than relying just on the kinetics of losartan disappearance, process efficiency should be assessed using integrated toxicity assays and the degree of mineralization (TOC removal). One significant gap in the current literature is the absence of consistency in the toxicity assessment of treated effluents [104]. The technical, operational, and crucial features of the primary AOPs mentioned for losartan degradation in aquatic matrices are compiled in Table 7.

Hence, the comparative analysis shown in Table 8 demonstrates that the efficiency of degradation of the parental compound is not the sole factor in selecting AOP to remediate the losartan. There exists a technological trade-off; the traditional Fenton process, which has lower reagent costs, also has greatly increased secondary environmental liabilities (i.e., production of ferrous sludge and need for pH adjustment). In contrast, processes (e.g., ozonation and activated persulfate) with high kinetics and selectivity have much higher labor and operational costs, and potentially greater risk of creating specific types of by-products, such as bromates and sulfonated compounds. In addition, the table illustrates that there is a major methodological gap in the current literature, as most published studies use analytical methods to estimate the degree of losartan elimination; however, little effort has been made to understand the ecotoxicity and mineralization of losartan transformation by-products. Given that oxidized transformation products will typically be more polar and bioavailable than the original matrix, their presence may lead to a greater acute toxicity to the final treated effluent compared to the original matrix; therefore, this fragmented approach to evaluating the performance of AOP may result in false positive results regarding their efficacy [105]. The results of the analysis demonstrate that no one treatment method has an ideal (or ‘optimal’) method of operation across all types of water bodies from the perspective of both sustainability and practical applicability. The quality of wastewater has a large impact on the technical feasibility of all AOPs utilized for removing pollutants; therefore turbidity, alkalinity (i.e., pH), as well as the extent to which free-radical scavengers are present in the water supply can greatly reduce the amount of pollutant removed at laboratory conditions. A major trend developing from this research is the need for multi-criteria evaluating criteria to be developed that consider removal efficiency, energy usage, waste produced during treatment, residual toxicity of the pollutant after treatment, along with the combined use of different forms of technology (synergism). Because of this, it will be necessary to change from the paradigm of reducing pollutants to one of ensuring that technologies used to reduce pollutants are ecologically safe in accordance with new technological innovations related to green chemistry and the circular economy in order to implement tertiary treatment processes for resistant pharmaceutical products such as losartan [104,106].

### 6.2. Photocatalysis

Heterogeneous photocatalysis is an emerging possibility for degrading emerging contaminants [107] due to its ability to use light energy to excite semiconductors and produce electron–hole pairs (e^−^/h^+^), which generate reactive oxygen species. The method offers the potential for complete mineralization of losartan, a drug that is highly chemically stable, using solar irradiation instead of artificial UV light or ozone generation with electricity, ultimately avoiding dependence on fossil fuels [108]. However, the efficiency of this process is limited by the kinetics of charge transfer at the solid–liquid interface and the optical properties of the photocatalyst. TiO_2_, in particular the anatase and rutile crystalline phases, is a preferred choice for a photocatalyst because of its chemical stability, low cost and lack of significant photo corrosion. Particular research on the photodegradation of losartan shows that TiO_2_ under UV light is efficient in cleaving the tetrazole group and the imidazole ring, which are essential structures for the compound’s pharmacological activity [109,110]. The primary critique, however, is on TiO_2_’s large energy bandgap (~3.2 eV), which limits its activation to the UV part of the spectrum (only ~4% of sunlight). In parabolic compound solar reactors, the quantum efficiency substantially drops in direct sunlight, necessitating longer hydraulic residence durations. Furthermore, the fast recombination of (e^−^/h^+^) pairs in pure TiO_2_ limits the production of OH radicals required for the breakdown of resistant intermediates by competing with surface oxidation processes [111].

Bandgap engineering techniques using metal or non-metal doping have been thoroughly studied to circumvent the spectrum constraint of TiO_2_ [112]. The introduction of intra-bandgap energy levels increases the ability of photocatalytic efficiency to degrade drugs such as losartan through solar photocatalysis. For example, studies show that nitrogen-doped TiO_2_ absorbs light up to approximately 550 nm. However, there are trade-offs because metallic dopants may leach into the effluent, creating secondary toxicity, and photo corrosion causes deactivation of the catalyst. Additionally, high dopant concentrations can serve as sites for charge recombination, which lowers the process’s overall efficiency. For ongoing operation in wastewater treatment facilities, the long-term durability of these changed materials in intricate aquatic matrices is still unclear [113].

An improvement in charge separation is shown by the incorporation of TiO_2_ with carbonaceous materials including graphene, CNTs, and biochar. The carbon structure in these composites serves as an electron acceptor, prolonging the life of charge carriers and postponing recombination (e^−^/h^+^). Losartan molecules are concentrated close to the semiconductor’s active sites by the high surface area and adsorption capacity of carbonaceous materials, which promotes surface oxidation through a “adsorption–degradation” process. The procedure is in line with the circular economy’s tenets by using biochar, which is made from biomass waste. However, the manufacture of composites using graphene or carbon nanotubes is expensive and requires complicated techniques. More importantly, the unintentional release of carbon nanoparticles into the aquatic environment poses ecotoxicological concerns that still lack explicit laws and long-term effect studies, and isolating the nanometric catalyst from the treated water is an ongoing engineering challenge [114,115]. In conclusion, even though photocatalysis has a strong theoretical potential for eliminating losartan, the challenges of catalyst recovery (in slurry systems) and the requirement for immobilization on stable supports without sacrificing photochemical activity prevent it from being implemented on an industrial scale. The environmental effects of nanomaterial manufacturing and the energy consumption of artificial lighting when solar irradiance is inadequate sometimes outweigh the benefits of pollutant degradation, according to life cycle assessments of these technologies. To ensure that the transformation byproducts are no more dangerous than the original medication, integrated toxicity testing should be conducted concurrently with implementation [116]. The technical features, processes, and crucial elements of the primary photocatalysts mentioned for the remediation of losartan in aquatic matrices are compiled in Table 8.

Advances in photocatalyst engineering for losartan degradation operate under a basic trade-off between photochemical efficiency and operational viability, as demonstrated by the comparative analysis displayed in the above table. Although pure TiO_2_ is stable and inexpensive, the use of solar irradiance is severely limited by its spectrum confinement to the UV region, which compromises quantum efficiency in large-scale systems. Doping and composition with carbonaceous materials are examples of modification techniques that increase absorption into the visible spectrum and enhance charge separation, but they also bring significant complications. For example, leaching metallic dopants can result in secondary toxicity, and the synthesis of composites containing graphene or carbon nanotubes raises costs and complicates nanomaterial recovery. The difference between the high analytical removal of losartan (>90% under optimum circumstances) and the sometimes incomplete mineralization (40–90% TOC removal) is more worrisome since it suggests that the treated effluent may still contain possibly more polar and ecotoxic transformation intermediates [116]. The use of these technologies in the environment is seriously jeopardized by this gap.

Table 8 emphasizes that no single photocatalyst offers an optimal profile for every aquatic matrix from the standpoint of sustainability and technological scaling. The unique difficulties associated with the matrix influence of the effluent and solution properties, cemented support generation, and other unsuccessful experimental designs may cause the reported effectiveness from laboratory-scale studies of benchtop pollution control to be much lower than 20–40% of the aforementioned percentages, which were obtained under conditions that differ from those used in typical laboratory-scale experiments [117]. The retention of the effective surface area and mass transport characteristics of the catalyst remains a major engineering hurdle for gel-based systems, while the retention of the catalyst in a liquid/slurry system will continue to be another significant engineering hurdle. As such, LCA can provide important inputs to the technology development process; however, to date, biochar composite materials that use waste streams as feedstock still need to be shown to have very low releases of nanoparticles and have suitable mechanical stability during use in continuous flow reactors [118]. The degree to which using photocatalysis for remediation of losartan solutions will exacerbate environmental problems caused by such technologies needs to be assessed in terms of not only the efficiencies at which they result in complete degradation of losartan, but also inclusion of multiple criteria, including degradation efficiencies, byproduct generation efficiencies, energy usage efficiencies, and residual ecotoxicological impacts.

### 6.3. Advanced Electrochemical Processes

Advanced electrochemical processes are a versatile group of remediation technologies that can achieve very specific applied voltages and may generate oxidizing agents in situ without the need to continually add outside chemicals. The potential for electrochemical reactions to allow for the adjustment of selectivity and oxidation intensity as it relates to the compound losartan is advantageous due to the extensive knowledge of the challenges of this compound and its persistence when treated through traditional biological remediation methods via the selection of electrode material and the operational parameters (current density, pH, and electrolytic matrix) associated with the electrochemical technology. The application of these technologies requires consideration of the energy requirements, the long-term stability of the electrodes, and the important consideration with respect to the characteristics of the products resulting from the transformation of the compound during the electrochemical oxidation [119]. In electro-oxidation, the pollutant can be oxidized directly at the anode surface or indirectly via the generation of reactive species through electrochemical oxidation (•OH, ozone, hydrogen peroxide, and active chlorine if used in a matrix that contains chlorides). Anodes with a lower oxygen overpotential (Platinum, Lead Dioxide, DSA) will generally promote partial oxidation or the generation of halogenated products, whereas anodes with a higher oxygen overpotential such as BDD will tend to produce physisorbed hydroxyl radicals (•OH_ads_) with high mineralization potential [120].

Research conducted on the electrochemical degradation of losartan shows that BDD is especially effective at cleaving the tetrazole moiety and imidazole ring of the molecule, with reductions of more than 95% being possible under optimal experimental conditions. However, a critical evaluation shows that there are several substantial limitations associated with the use of BDD or other advanced anodes in large-scale applications: (i) the cost of BDD and the other advanced anode materials limits their application to only small-scale projects; (ii) in the presence of chloride ions, the oxidative mechanism shifts to the generation of free chlorine (as in the oxidation of chloride ions), which leads to the generation of organochlorine byproducts that could be more toxic and persistent than the original drug [121]; (iii) when a supporting electrolyte must be added to low conductivity effluents, the energy required for complete mineralization (>80% TOC removal) continues to be significant. Additionally, with time, fouling of the electrode surface by organic waste or polymeric intermediates can lower performance, necessitating regular cleaning procedures or electrode replacement [122].

Researchers analyzed the electrochemical oxidation of losartan, an emerging pharmaceutical pollutant [123]. Using a BDD/stainless steel system, electrochemical oxidation was carried out in batch mode in a 100 cm^3^ open, undivided cell. In experiments using SO_4_^2−^ as a supporting electrolyte, a greater mineralization yield of 67% was obtained, including the whole elimination of potassium losartan at 80 mA cm^−2^ and a reaction duration of 180 min at pH 7. In contrast, 56% mineralization was observed with a medium containing Cl^−^. The degree of mineralization and the effectiveness of the electrochemical oxidation process both increased with higher potassium losartan concentrations. UHPLC-MS/MS was used to identify up to four aromatic intermediates during mineralization. Moreover, ion-exclusion HPLC was used to identify and measure short-chain and linear carboxylic acids, including oxalic, succinic, and oxamic acids. Lastly, at ideal conditions (10 mA cm^−2^ 0.05 M Na_2_SO_4_, pH 7, 25 °C, and 360 min of electrolysis), the electrochemical oxidation method was able to mineralize dissolved commercial tablets containing losartan, achieving TOC removal of up to 71% [123].

**Figure 4:**
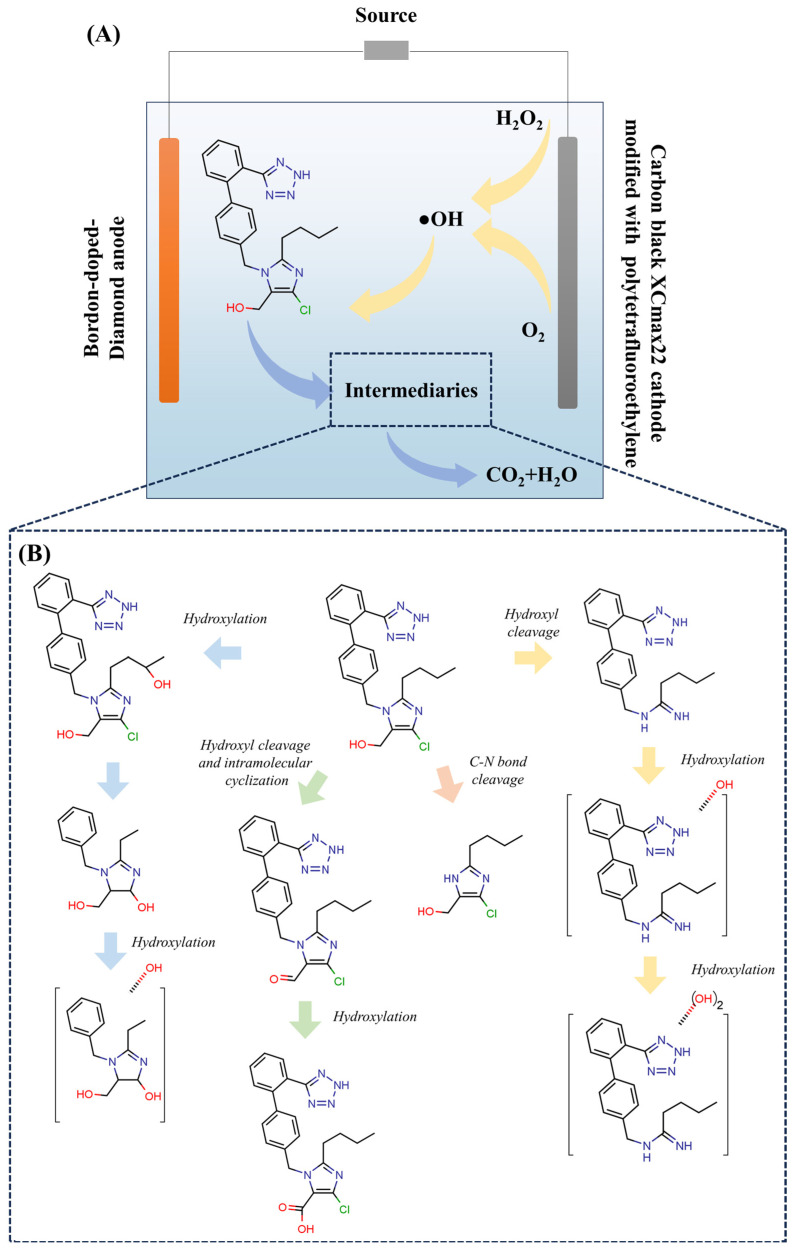
(**A**) Representation of the losartan electrochemical oxidation process; (**B**) Proposed degration path for the losartan.

Another study found that employing hydrogen peroxide generated in situ during electrochemical oxidation processes increased the elimination of losartan, irbesartan, and related transformation products (Figure 4) [90]. An electrochemical cell with a BDD anode and a carbonaceous cathode consisting of cross-linked glassy carbon was used to study the degradation of losartan and irbesartan. The cathode modified with two types of CB (XC72 and XCmax22) showed in situ H_2_O_2_ generation. The cathode built with XCmax22 at a mass ratio of 1/4 relative to PTFE (i.e., PTFE/CB) produced the most effective H_2_O_2_. Overall, XCmax22 outperformed XC72, generating nearly twice as much H_2_O_2_ and reaching a current efficiency of up to 93%. Additionally, the electro-oxidation rate of losartan and irbesartan was greatly accelerated by in situ created H_2_O_2_, resulting in an almost six-fold faster elimination of both compounds. An initial concentration of 10 mg L^−1^ of each drug was eliminated in 10 min. Furthermore, the impact of the aqueous matrix was examined at a modest starting concentration of the chemicals (500 ng L^−1^). In total, 500 ng L^−1^ of both compounds was completely removed in 30 min due to the compounds’ pseudo-first-order rates of degradation. The transformation products of losartan and irbesartan were identified, the degradation pathways were suggested, and their toxicity was assessed using ultra-high performance liquid chromatography in conjunction with high-resolution mass spectrometry (UHPLC-HRMS). These findings provide a method for the conversion and storage of excess electrical energy in the form of chemical energy (i.e., H_2_O_2_) as well as an economical and energy-efficient approach for the treatment of resistant organic micropollutants [90].

The EF process is an extension of the conventional Fenton process, wherein the ferrous catalyst is electrochemically regenerated (Fe^3+^ + e^−^ → Fe^2+^) and hydrogen peroxide is produced in situ by the biphasic reduction of oxygen at the cathode (O_2_ + 2H^+^ + 2e^−^ = H_2_O_2_). Because iron is recycled in the catalytic cycle, this arrangement lessens two significant drawbacks of the homogeneous Fenton process: the requirement for constant H_2_O_2_ addition and the buildup of ferric sludge [124]. Because of the continuous production of OH radicals in bulk solution, EF exhibits faster degradation kinetics for losartan than traditional electro-oxidation [125]. The electro-Fenton technique has benefits, but there are operational issues that need be carefully considered. First, the process’s ideal pH is still limited to the acidic range (2.5–3.5), necessitating pre- and post-treatment modifications that complicate operations and produce secondary saline effluents. Second, the matrix affects the effectiveness of cathodic H_2_O_2_ generation: the creation of oxidizing species can be severely hampered by the presence of radical scavengers (bicarbonate, natural organic matter) or competitors for oxygen reduction. Thirdly, without methods for immobilizing iron in conductive matrices (such as cathodes modified with chelating agents), the system’s reusability is limited by the stability of the ferrous catalyst in continuous flow systems and the potential precipitation of hydroxides at neutral pH [126]. Lastly, similar to other AOPs, losartan’s partial degradation can produce transformation intermediates that are highly ecotoxic [127].

In this regard, ARBs, valsartan and losartan, which are used to treat hypertension and are regarded as emerging contaminants, were degraded using PEF processes employing a DSA-GDE under light-emitting diode radiation [128]. The objective of investigating the use of citric acid, tartaric acid and oxalic acid as iron ion complexation agents was to maintain the Fenton reaction environment at a pH close to neutral. Data suggest that after 90 min of EF therapy, 70% of valsartan and 100% of losartan were degraded at a current density of 3.42 mA m^−2^. Complete degradation of both losartan and valsartan and 30% mineralization of these compounds were observed using the PEF process. Using citric or tartaric acid as iron ion complexation agents resulted in the same degree of degradation (100%) of losartan and valsartan in 90 min as did the use of oxalic acid. The increasing amount of initial dissolved iron within the system will promote the conversion of Fe^3+^/Fe^2+^ in the catalytic photo-Fenton reaction, and, therefore, the production of hydroxyl radicals. Furthermore, the enhanced capacity of the complexes to complex Fe^3+^ and provide ligand-to-metal charge transfer, which are critical for the provision of Fe^2+^ to the Fenton process, may explain their increased photoactivity. The findings demonstrate that, as compared to conventional UV-A lamps utilized in this kind of work, the assessed system was more effective at removing chemicals from the sartan family using LED light. Additionally, HRMS identified three transformation products from the degradation of valsartan and two from the degradation of losartan. Following the PEF system, the different accumulated and non-mineralized organic compounds were successfully treated in a subsequent aerobic biological system [128].

**Figure 5:**
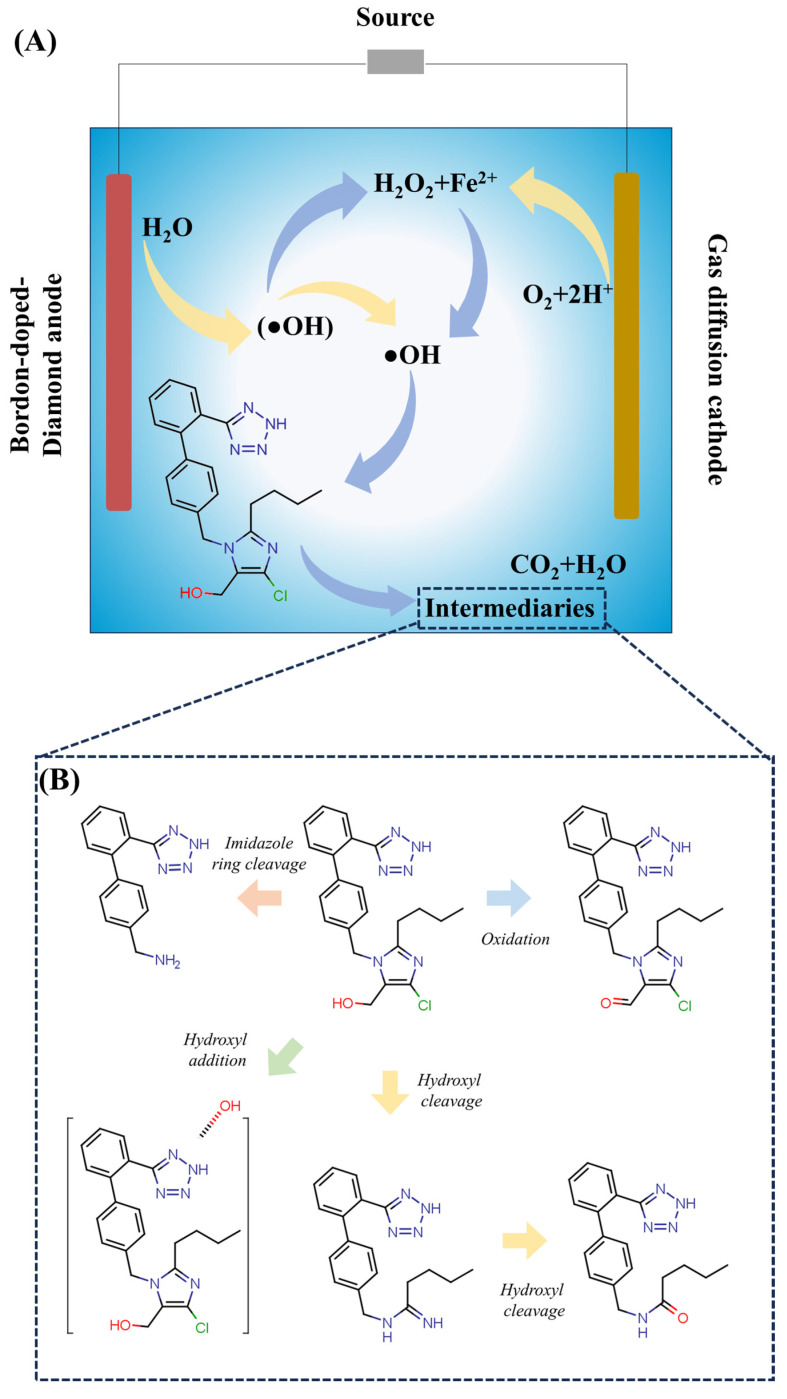
(**A**) Electrochemical cell setup with BDD anode and gas diffusion cathode facilitating hydroxyl radical production; (**B**) suggested transformation products and reaction mechanisms involved in the electrochemical degradation of losartan.

In a different investigation, losartan antihypertensive medications were treated in water utilizing photoelectro-Fenton with BDD anodes (Figure 5). These contaminants were quickly eliminated by PEF (>95% in 30 and 60 min of treatment for losartan and valsartan, respectively) [129]. With pseudo-first-order rate constants of 0.154 and 0.054 min^−1^ for losartan and valsartan, respectively, the attack by electrogenerated radicals was the primary elimination mechanism. To explain the antihypertensives’ reactivity to the electrogenerated degrading agents, theoretical atomic charge analyses were carried out. The primary transformation products were then assessed. The transformation products showed that the degrading species target the alcohol, imidazole, and biphenyl-tetrazole groups in losartan. But in valsartan, the core nucleus was altered along with the amide and carboxylic groups. These sections matched the electron-rich regions found in the theoretical simulations quite well. Additionally, after five hours of electrolysis, the PEF procedure eliminated between 33 and 38% of the total organic carbon. Lastly, the degradation of the pollutant in effluents from municipal wastewater treatment plants by PEF at pH ∼5 was taken into consideration, as was the treatment of losartan in the presence of oxalic acid, a common organic remnant from the pharmaceutical industry. Losartan was broken down more quickly by oxalic acid. After 120 min of treatment, 64% of the losartan in the effluent was removed, demonstrating the strong potential of PEF to break down antihypertensive medications in water that contains both organic and inorganic chemicals [130].

In summary, electro-oxidation and electro-Fenton processes have excellent technical promise for remediation of losartan; however, their feasibility depends on addressing several barriers related to the environment (byproduct management and residual toxicity) and to economy (cost of advanced electrodes and energy use). To simultaneously achieve an acceptable degree of degradation efficiency, operating cost, and ecotoxicological safety, hybrid electrochemical approaches combined with biological methods or separation membranes may be effective [130]. The technical features, processes, and crucial elements of electro-oxidation and electro-Fenton used for losartan remediation in aquatic matrices are compiled in Table 9.

### 6.4. Emerging Adsorbents and Nanomaterials

**Figure 6:**
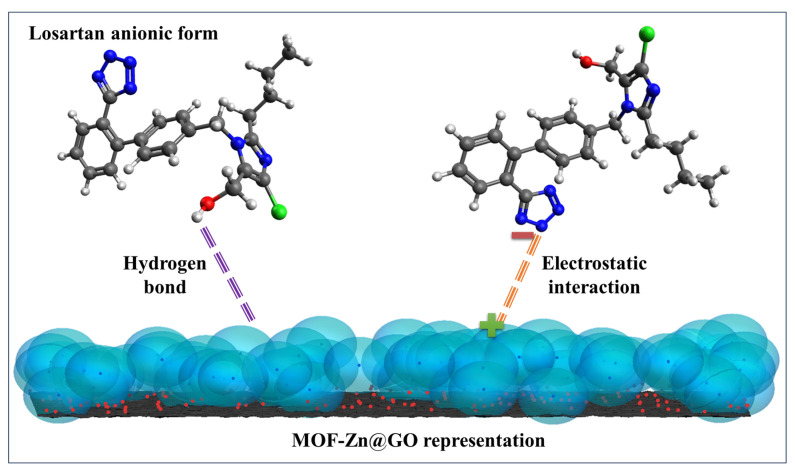
Schematic representation of the adsorption mechanism of the Losartan anion onto the MOF-Zn@GO surface, highlighting hydrogen bonding and electrostatic interactions.

Adsorption remains a cornerstone strategy for losartan removal, shifting the contaminant from the aqueous to the solid phase rather than destroying it [131]. Recent advances have focused on developing materials with tailored porosity, surface functionality, and magnetic separability to overcome the limitations of conventional activated carbon, particularly given losartan’s aromatic structure and ionizable tetrazole/imidazole groups [132,133]. Biochar, derived from agro-industrial residues, offers a cost-effective and circular alternative, relying on π–π interactions, pore filling, and hydrogen bonding. However, its structural heterogeneity, potential leaching of residual compounds, and rapid saturation in complex matrices hinder standardization and necessitate energy-intensive thermal regeneration [5,134]. For instance, sugarcane bagasse-derived biochar has demonstrated rapid kinetics and high removal efficiency (~99.8%) across diverse pharmaceuticals in real effluents, effectively mitigating ecotoxicological risks while maintaining affordability and ease of handling [135]. Graphene-based materials (GO/rGO) exhibit exceptional surface areas and strong π–π stacking with losartan’s biphenyl-tetrazole core, achieving capacities of 100–350 mg g^−1^ [136]. Nevertheless, their high synthesis cost, tendency to aggregate in saline waters, and risks of nanosheet leaching remain critical barriers. Hybridizing GO with MOFs (e.g., MOF-Zn@GO) enhances structural stability and yields high capacities (~395 mg g^−1^) through synergistic hydrogen bonding and π–π interactions, maintaining >85% efficiency over five cycles (Figure 6) [5].

**Figure 7:**
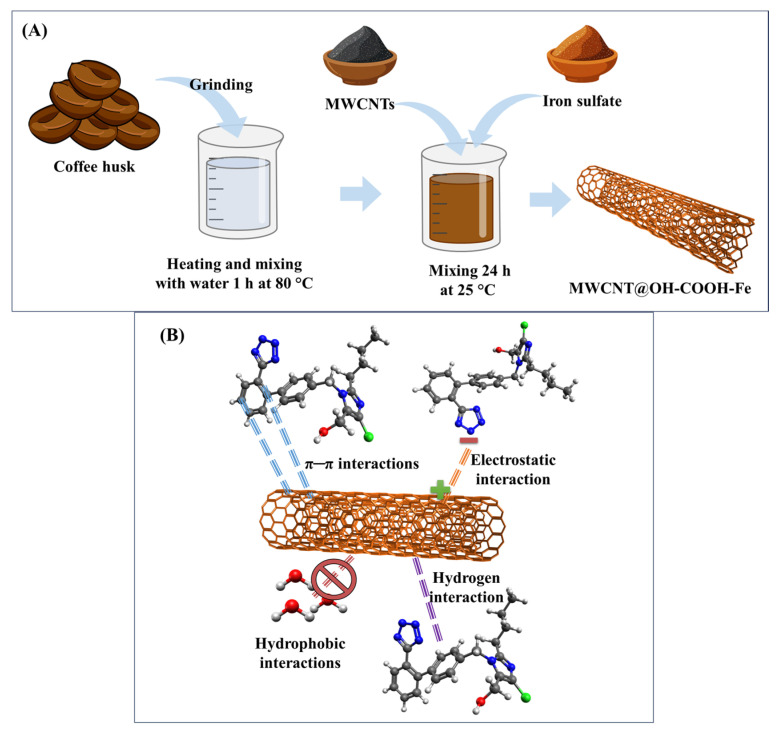
(**A**) Environmentally friendly synthesis route of MWCNT@OH-COOH-Fe adsorbent derived from coffee husk; (**B**) molecular interactions governing the uptake of Losartan, including π–π stacking, electrostatic attraction, and hydrogen bonding.

To remove losartan from aqueous solutions, researchers have suggested using an adsorbent made of multi-walled carbon nanotubes functionalized with iron nanoparticles, which were produced using an environmentally friendly method (Figure 7) [137]. Within the investigated temperature range, the results showed constant maximum adsorption capabilities (q_LOS_ = 0.534–0.569 mmol g^−1^; q_DIC_ = 0.539–0.559 mmol g^−1^). Concurrent mechanisms such as π–π, n-π, hydrogen, electrostatic, and hydrophobic interactions are involved in adsorption. The given results provide a sustainable option for the removal of developing pollutants from aqueous solutions, as well as to further our understanding of adsorption processes [137].

**Figure 8:**
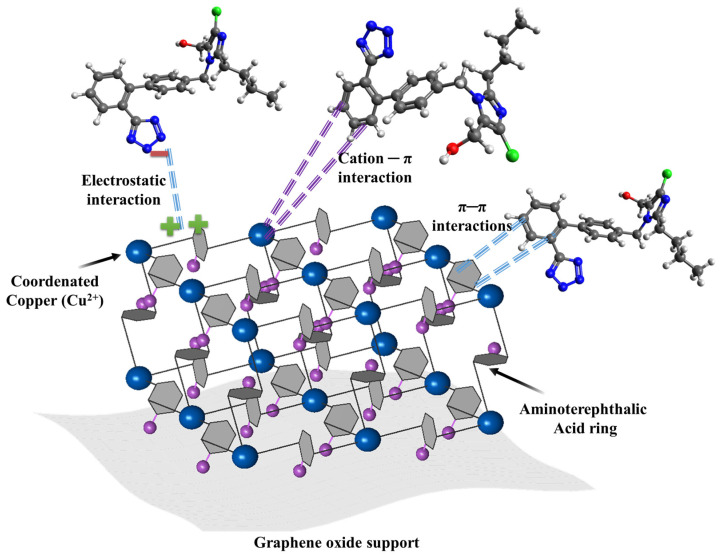
Molecular model of the MOF-Cu@GO structure showing the coordinated copper ions (Cu^2+^), aminoterephthalic acid rings, and graphene oxide support, illustrating the key pathways for losartan.

One other study investigated using a new structure for a copper(II)-based MOF that is impregnated with graphene oxide (MOF-Cu@GO) for the purpose of using it as an efficient microphase dispersion solid phase extraction (D-μSPE) material for the removal of losartan from water [138]. The MOF-Cu@GO sorbent demonstrated an adsorbent capacity for losartan of 415 mg g^−1^. The hypothesized mechanism by which losartan molecules were adsorbed to MOF-Cu@GO was through hydrogen bonding, unsaturated ligand sites, π–π interactions, and through electrostatic attractions (Figure 8) [138].

MOFs’ have high porosity and chemical tunability, and cavities tailored to losartan’s size and molecular shape can be created. In MOFs like ZIF-8 or UiO-66, coordinative interaction and spatial confinement have shown encouraging removal efficiencies. However, their hydrolytic instability in water and metal ion leaching at extreme pH remain critical drawbacks [139]. To mitigate particle aggregation and structural collapse, hierarchical composites like ACNF/MIL-68(In)–NH_2_ have been developed, where acid-treated carbon nanofibers serve as a 3D scaffold to distribute MOF nanoparticles uniformly, significantly boosting active site accessibility and porosity [140]. The resulting ACNF/MIL-68(In)–NH_2_ composite achieves rapid equilibrium (~64 min) with experimental capacities >250 mg g^−1^ and theoretical maxima >630 mg g^−1^. Driven by electrostatic attraction, hydrogen bonding, and π–π stacking, this material effectively removes trace losartan even in the presence of competing ions, demonstrating strong potential for continuous-flow municipal wastewater applications [83].

**Figure 9:**
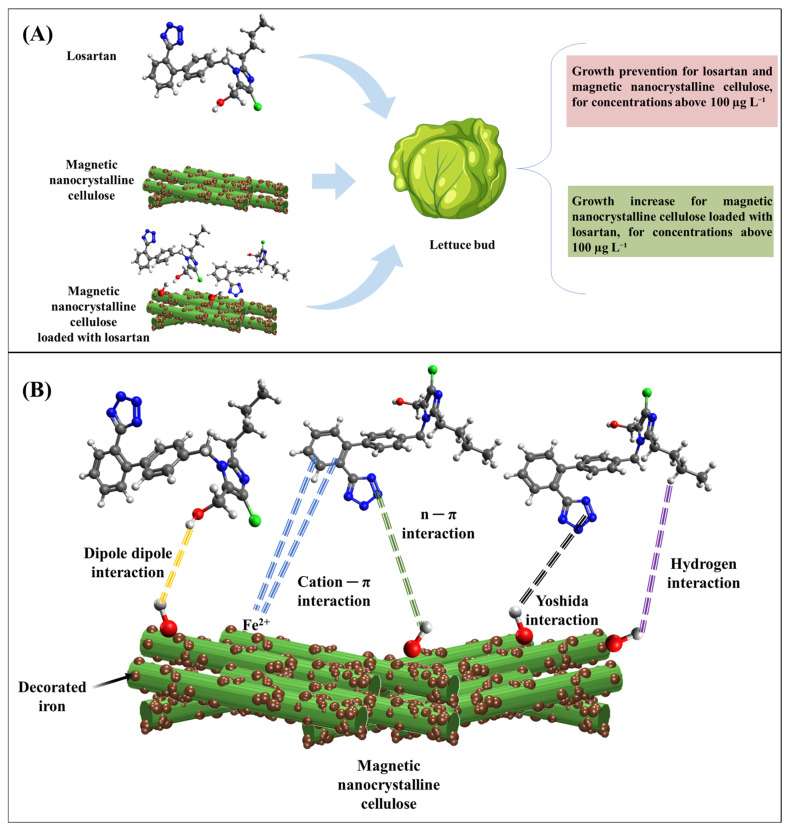
(**A**) Phytotoxicity assay results showing the protective effect of the loaded adsorbent on lettuce growth compared to free losartan; (**B**) proposed adsorption mechanisms on magnetic nanocrystalline cellulose, highlighting specific interactions such as hydrogen bonding, cation–π, and dipole interactions.

Functional biopolymers and magnetic nanocomposites combine biodegradability with easy magnetic recovery. Nanocellulose, derived from renewable sources, offers high surface area and mechanical strength, while magnetic nanoparticles (Fe_3_O_4_) enable rapid separation and reduce operational complexity [141,142,143] In this regard, a study used an effective and eco-friendly method to remove losartan from the aquatic environment through adsorption by combining a biodegradable and magnetically recoverable cellulose–magnetic nanocomposite (NC·Fe_3_O_4_) with a thorough evaluation of adsorption efficiency and post-adsorption safety profile (Figure 9) [144]. For this, the virgin adsorbent, the losartan and adsorbent loaded with losartan were tested to evaluate their influence on the *Lactuca sativa* (lettuce) growth. According to the authors, the plant was able to grow normally with the presence of virgin adsorbent, with a concentration of 10 and 100 µg L^−1^, and bud size of 3 to 5 cm. However, increasing the virgin adsorption up to 1000 µg L^−1^ prevented the plant growth, directly diminishing the germination and radicle elongation. The same trend was also reported for losartan. For the losartan-loaded adsorbent, ecotoxicological assays demonstrated a mitigating effect at concentrations above 100 µg L^−1^, indicating that the adsorbent alleviates losartan phytotoxicity through molecular immobilization and controlled release, thereby reducing its environmental bioavailability. The NC·Fe_3_O_4_ nanocomposite achieves ~96% removal at pH 4 and maintains efficiency over six cycles [144].

According to Ijaz et al. [145], the adsorption of indomethacin and losartan onto this new material (DTT@BC@MoBTx) was positively correlated with the amount of functional groups, the surface area the particular adsorbent had, and delivery of greater porosity. The performance of DTT@BC@MoBTx was exceptional given its highest sorption (or adsorption) capacities for indomethacin (1041.26 mg g^−1^) and losartan (887.31 mg g^−1^). After performing repeat adsorption–desorption tests (n = 4), there was a small decrease in the ability of losartan and indomethacin to be adsorbed onto the adsorbent. The clearance rates for both losartan and indomethacin were minimally influenced by monovalent ions (K^+^, Na^+^, NO_3_^−^, and Cl^−^) at each concentration tested. Additionally, ionic species with higher valence states (Ca^2+^, Mg^2+^, CO_3_^2−^, and SO_4_^2−^) are more likely to interfere with the adsorptive capacity for losartan and indomethacin than lower valence ions (K^+^, Na^+^, NO_3_^−^, and Cl^−^) [145]. Montmorillonite clay has an exceptional capacity for expanding and exchanging cations, which makes it a viable candidate for use as an adsorbent. It is widely used to remove pollutants due to its abundance, low cost and ease of modification. A potential organoclay that emerged from the research was Spectrogel–Type C (or Spectrogel), a commercially produced Brazilian organoclay with dialkyl dimethylammonium as the exchanging cation [92]. This research is the first to ever use clay minerals to assess the adsorption of losartan. The effect of the initial pH of the solution (2.5 to 10) on the experimental process was not significant; however, 6 g L^−1^ exceeded all other adsorbent doses for between the two types of adsorbents [92].

Recent investigations have also explored the use of microplastics as unconventional adsorbent matrices for pharmaceutical retention. In controlled batch assays, polystyrene and polyethylene particles demonstrated a measurable affinity for losartan, with adsorption reaching ~59% after 168 h under static conditions. The interaction is primarily driven by hydrophobic partitioning and π–π stacking between the drug’s aromatic rings and the polymer surface, rather than electrostatic forces. Subsequent coagulation–flocculation trials using iron- or manganese-based coagulants were not intended to directly remove dissolved losartan, but rather to aggregate and settle the contaminant-loaded microplastic particles, achieving removal rates of 72–92% for the solid phase. While microplastics are not proposed as engineered adsorbents, these findings highlight their unintended role as secondary vectors for pharmaceutical transport in aquatic systems and underscore the need to evaluate adsorption-mediated co-transport mechanisms in complex wastewater matrices [77].

As systematically compiled in Table 10, the performance of emerging adsorbents is governed by fundamental trade-offs that dictate their scalability. While graphene and MOF-based composites offer the highest reported capacities (up to 500 mg g^−1^) and rapid kinetics (equilibrium in 10–60 min), their synthesis costs, sensitivity to ionic strength, and regeneration losses restrict them to niche or polishing applications. In contrast, biochar and organoclays present lower upfront costs and broader pH tolerance, but require frequent replacement or thermal regeneration. Magnetic nanocellulose and functionalized biopolymers strike a balance between moderate capacity (15–120 mg g^−1^), easy separation, and low ecotoxicity, yet their mechanical swelling and limited reusability remain unresolved. Crucially, Table 10 highlights a consistent gap across all material classes: the absence of standardized protocols for assessing long-term stability in dynamic wastewater matrices, the environmental fate of spent adsorbents, and the real energy footprint of regeneration cycles. Bridging this gap requires shifting from batch-capacity benchmarks to continuous-flow performance metrics and life cycle-informed material selection. In conclusion, while emerging adsorbents outperform conventional materials in laboratory settings, their real-world deployment is constrained by secondary waste management, regeneration energy demands, and the lack of continuous-flow validation. Future efforts must prioritize standardized synthesis, long-term stability in real effluents, and closed-loop regeneration strategies to align adsorption with circular economy principle.

### 6.5. Hybrid Technologies

The challenging structure and pseudo stability of Losartan in the environment illustrate how all technologies developed independently will have limiting factors related to the efficacy of removal and total mineralization as well as potential for ecotoxic effects. In this scenario, hybrid technologies are intuitive strategies that utilize two or more methods together to create synergistic effects in an attempt to overcome the kinetic and operational limitations associated with each individual technology. The fundamental principle behind hybrid technologies is one of the complementarity of mechanisms whereby one of the technologies will encourage the transformation or concentration of the pollutant and another will provide for the final mineralization or physical separation of the pollutant with the intent to optimize the trade-off between sustainability, cost and efficiency [146]. In order for hybrid systems to be implemented, an extensive examination of the operational compatibility between different processes and how they generate intermediate byproducts as well as their ability to be scaled up must take place. One possible hybrid method for disposing of medications that are resistant to normal degradation, such as losartan, is to combine AOPs with a biological treatment. AOPs such as ozonation, the electro-Fenton process or photocatalysis can be used as a pretreatment to partially convert a medication into more biodegradable products, such as hydroxylated compounds and short-chain carboxylic acids, prior to its complete mineralization by microorganisms in a biological reactor (e.g., biofilters, MBBR or MBR). Studies have shown that if ozonation is performed at controlled doses (0.5–1 g O_3_ g^−1^ TOC), it increases the potential for the losartan-containing effluent to undergo complete degradation (a BOD⋅/TOC ratio greater than 0.4), thus resulting in more than 95% removal of TOC from the final integrated system [147]. A critical review indicates also that (i) it is necessary to control the oxidative dosage, as high levels may produce toxic and/or recalcitrant by-products which may inhibit microbial activity; (ii) microbial growth and the rate of organic degradation via AOP must be in agreement to avoid hydraulic or organic load imbalances; and (iii) oxidizing agents, such as residual hydrogen peroxide or residual ozone in AOP effluent, may require a neutralization step prior to entering a biological reactor [148]. Also, due to the fact that oxidized intermediates may present short periods of ecotoxicity prior to completely biologically mineralizing, it is important to measure toxicity over the whole treatment chain [116].

The incorporation of membrane separation techniques (ultrafiltration, nanofiltration, and reverse osmosis) and adsorbents results in a hybrid technique that achieves target chemical capturing through chemical removal and selective physical removal. The adsorbent can be used by either (1) slurring the adsorbent in the reaction medium (adsorptive membrane filtration processes) or (2) employing the adsorbent in a fixed bed prior to the membrane. The adsorbent can also be used to compete with natural organic matter, thereby preferentially holding onto losartan and inhibiting membrane fouling. According to research, magnetic biochar and nanofiltration membranes may remove >98% of losartan while reducing fouling by 40–60% when compared to standalone membranes [149]. However, the following operational constraints need to be carefully considered: (i) the addition of particulate adsorbents can increase the medium’s viscosity and transmembrane pressure, which increases energy consumption; (ii) simultaneous adsorbent saturation and membrane fouling require complicated regeneration protocols, which are frequently incompatible with one another (chemical desorption of the adsorbent vs. physical cleaning of the membrane); and (iii) leaching of adsorbent nanomaterials can contaminate and lower the quality of the final effluent [150]. These difficulties are somewhat mitigated by techniques like immobilizing the adsorbent on the membrane surface or employing recoverable magnetic materials, but they increase the hybrid system’s synthesis complexity and expense [151].

**Figure 10:**
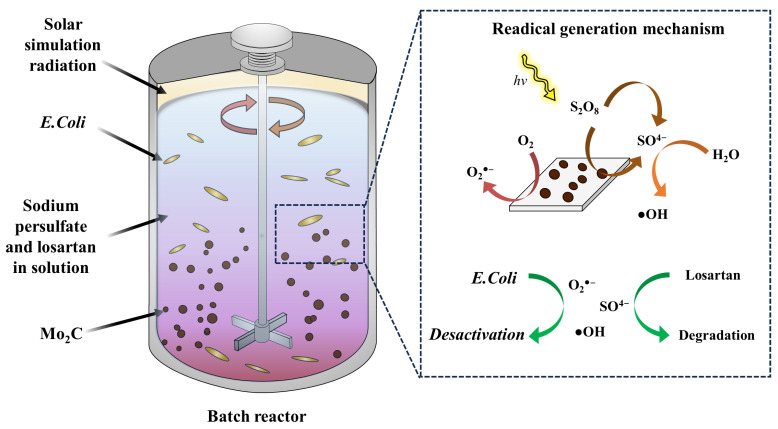
Schematic illustration of the Mo_2_C/peroxydisulfate process showing the radical generation mechanism and the pathways for losartan removal and bacterial disinfection.

Through integration with additional oxidative or separation processes, coupled photocatalysis systems seek to overcome the constraints of pure TiO_2_’s quantum efficiency and selectivity, such as photocatalysis + persulfate, where the photocatalytic activation of persulfate produces sulfate radicals (SO_4_^−^) with higher selectivity and half-life than OH^−^; (i) PEC, which combines photocatalysis with applied electrical potential to improve charge separation and generate additional oxidants; and (ii) photocatalysis + membrane, where the catalyst is immobilized on membrane supports to facilitate recovery and promote continuous surface oxidation [152]. The efficacy of a Mo_2_C/peroxydisulfate system for treating tertiary wastewater containing losartan was examined (Figure 10) [153]. In less than 45 min, 500 μg L^−1^ of losartan was broken down using 500 mg L^−1^ of Mo_2_C and 250 mg L^−1^ of Sodium Persulfate (SPS). The apparent kinetic constant dropped as the concentration of losartan increased; however, the breakdown of losartan was accelerated at acidic pH. Experiments carried out in the presence of reactive species scavengers revealed that singlet oxygen, hydroxyl radicals, and sulfate radicals all contributed to the oxidation of losartan, with singlet oxygen being the most common reactive species. Interestingly, the inclusion of chloride increased the rate of degradation whereas competitors like bicarbonate and organic matter decreased the efficiency seen in real matrices. After five consecutive trials, the catalyst demonstrated exceptional stability, maintaining full losartan removal. The system’s ability to simultaneously break down losartan and eradicate *Escherichia coli* was assessed. Although the system was able to lower the *E. coli* content by 1.23 log, the presence of *E. coli* slowed the breakdown of losartan, resulting in only 30% elimination after 3 h. However, in the presence of simulated solar irradiation, losartan was entirely destroyed in 45 min and the *E. coli* concentration was reduced by about 4 log, indicating a considerable synergistic effect of the solar/Mo_2_C/SPS combination [153].

One of the most promising and ecologically benign methods for removing medications from aqueous media is solar-induced semiconductor photocatalysis. In this regard, a bismuth oxychloride (BiOCl) photocatalyst was created and its shape, crystallographic structure, and optical characteristics were described [154]. Using a solar simulator, its photocatalytic efficiency was evaluated in the breakdown of losartan. Significant photocatalytic efficiency was demonstrated by the produced BiOCl, which completely degraded 0.3 mg L^−1^ of losartan in brief irradiation times (15–30 min). Using pH 3 (k_app_ = 0.32 min^−1^) and 500 mg L^−1^ of BiOCl (k_app_ = 0.21 min^−1^), the system under investigation demonstrated maximal efficiency. However, in environmentally important aqueous matrices, such as wastewater (k_app_ = 0.006 min^−1^) and bottled water (k_app_ = 0.023 min^−1^), losartan removal dramatically decreased. Additional experiments on artificial aqueous matrices revealed that the presence of humic acid (k_app_ = 0.016 min^−1^) and bicarbonates (k_app_ = 0.029 min^−1^) decreased the rate of losartan degradation by more than 40%, but chlorides had no effect on overall efficiency. Moreover, the predominant oxidizing species were singlet oxygen and photogenerated holes. A pilot-scale flat-plate photoreactor was then used to investigate the effectiveness of the BiOCl photocatalyst in the degradation of losartan. It was discovered that after 100 kJ L^−1^ of cumulative sun irradiation, almost 75% of the losartan was eliminated. The pilot unit results validated BiOCl’s suitability as a possible photocatalytic material [154].

Despite their theoretical synergies, hybrid remediation systems face substantial barriers to real-wastewater implementation. Matrix interference remains the primary bottleneck: NOM, bicarbonates, and competing ions rapidly scavenge reactive species in AOP-coupled systems or foul membranes and adsorbent sites, often reducing laboratory-reported efficiencies by 40–70% in continuous flow. Operational complexity further limits scalability; hybrid trains require precise dosing control, real-time monitoring (e.g., UV_254_, DOC, redox), and multi-stage regeneration protocols that are rarely compatible or economically viable at municipal scales. Moreover, secondary pollution risks are frequently overlooked: AOP pretreatment can generate transiently toxic transformation products that inhibit downstream biological reactors, while membrane-adsorbent hybrids concentrate contaminants into brine or spent cartridges that lack standardized disposal pathways. Energy and chemical demands for hybrid configurations (e.g., UV/persulfate, electro-Fenton, or photocatalytic membranes) often exceed the operational budgets of decentralized or peri-urban facilities, particularly in regions with unstable energy grids. Critically, the literature lacks long-term pilot studies under variable hydraulic and organic loads, meaning most hybrid performance metrics are extrapolated from idealized synthetic waters. Until hybrid systems are validated through continuous-flow pilot trials, integrated techno-economic assessments, and full-scale matrix tolerance testing, their transition from bench-scale promise to municipal reality will remain constrained. Future research must therefore prioritize flexible, modular designs validated under dynamic real-world conditions, coupled with rigorous life cycle and ecotoxicological assessments to ensure economic and environmental viability.

## 7. Scientific Gaps

Despite a considerable amount of literature on the degradation of losartan in aquatic systems, there remain major gaps in the ability to translate laboratory experiment results into applications at full scale due largely to significant structural barriers. A technological optimism, which has been shown to not readily adapt to the dynamic and complex nature of environmental systems and wastewater treatment facilities, is created by the large volume of laboratory studies that have been conducted under highly controlled and ideal conditions (i.e., synthetic water, static batch reactors). This creates a disparity between analytical and environmental efficacy that will inhibit the future application of effective remediation technologies. A careful assessment and critique of these issues is essential in guiding scientific progress towards sustainable, commercially viable, and environmentally friendly solutions. One of the most critical limitations is the lack of long-term investigations to evaluate the durability and long-term operational stability of proposed materials and processes. Most studies on photocatalysis, adsorption and electrochemical processes provide data from short-term studies (from hours to days), and therefore, it will be difficult to predict the operational life of catalysts, membranes, and adsorbents under continuous operation conditions. In the case of losartan, there is insufficient consolidated information on the loss of performance of new materials after being tested over a number of months with real effluents, and their ability to be regenerated after repeated saturation and desorption cycles. Long-term economics and eco-friendliness can be affected when catalytic deactivation mechanisms, such as fouling, active site poisoning and leaching of structural materials, are underestimated. In addition, long-term tracking of any potential late release of retained contaminants or transformation products from the final environmental fate of created waste (sludge, spent adsorbers) is a necessary component of thorough life cycle analyses.

Most of the studies that study losartan removal are from Europe, North America and some regions in Asia, which shows a geographic bias in the literature. Developing countries, especially in Latin America and Africa, have not been well represented in this area. Access to basic sanitation in these countries varies greatly and there are likely fewer environmental laws. This is crucial because local pharmaceutical use patterns and disposal methods frequently result in wastewater matrices in these areas showing greater organic loads, unique conductivities, and particular pollutant profiles. In situations with significant energy and financial constraints, technologies validated in wastewater treatment plants in Europe might not be commercially viable or technically practicable. The development of context-specific solutions is hampered by the lack of local data, which increases the dangers to regional public health by maintaining reliance on imported technology that might not be able to adequately reduce losartan contamination in these areas.

One major obstacle is still the shift from bench-scale to pilot or industrial size. Pilot-scale investigations are uncommon and, when they do exist, show significantly lower efficiency because of uncontrolled matrix effects, despite the fact that analytical removal of losartan is commonly claimed to reach 90% in laboratory reactors. Predictive models seldom take into account the hydraulic complexity of continuous-flow reactors, the existence of NOM or adsorption sites, and the seasonal change in effluent composition. Moreover, comprehensive economic-technical analysis at full size is sometimes neglected or predicated on theoretical projections that fail to account for secondary waste management, material replacement, and maintenance expenses. Due to the lack of solid data regarding the true cost–benefit of applying cutting-edge technology for particular contaminants like losartan, public managers and WWTP operators are unable to make informed decisions.

The overemphasis on eliminating the parent compound at the expense of toxicological characterization of the TPs may be the most significant gap. A series of chemical intermediates are produced when losartan is broken down via AOPs or hybrid processes. Some of these intermediates may still have pharmacological function or be more ecotoxic than the original molecule. Systematic investigations of bioassay batteries in conjunction with the structural identification of hazardous contaminants using HRMS are lacking in the current literature. Often, the “efficiency” of treatment is determined just by the decrease in losartan concentration found by chromatography, neglecting the possibility that harmful or recalcitrant byproducts may occur and remain in the final effluent. Technologies that simply convert a known pollutant into a complex mixture of unknown contaminants run the risk of being validated without an integrated toxicity assessment based on consequences, endangering human health and ecological safety.

## 8. Future Prospects

Remediation of losartan in aquatic matrices is at a paradigm shift in which scientific knowledge now needs to be transformed epistemologically to facilitate the transition from proof-of-concept studies through to engineering-scale approaches. The development of high adsorption capacity materials or fast oxidation kinetics will not be the sole approach to future management of this emerging contaminant; rather all four components (technical efficiency, environmental sustainability, economic viability, regulatory sufficiency) must work together in concert. Future perspectives should focus on overcoming the traditional dichotomy between analytical performance and environmental effectiveness by placing an emphasis on operational robustness under real-world conditions, minimizing the carbon footprint associated with treatment processes, and ensuring evidence-based ecotoxicologically safe products. Due to the need of decarbonizing treatment technologies and encouraging the valorization of agro-industrial waste, the development of biomass-derived adsorbents (biochar, chitosan, and functionalized lignin) tends to consolidate as a key research field. To guarantee molecular selectivity for losartan in complex matrices, rational surface engineering should be the main emphasis of future research rather than only the empirical synthesis of novel biocomposites. Future research must incorporate cradle-to-grave life cycle assessments to confirm whether the chemical agents used in functionalization do not outweigh these materials’ inherent environmental advantages. Furthermore, in order to enable industrial replication and operational dependability, the physicochemical characteristics of biochars made from various biomasses must be standardized using harmonized processes. In order to maximize electrostatic interactions without sacrificing the adsorbent’s regenerability, future research should investigate selective functionalization with ionizable groups that take advantage of losartan’s pH-dependent speciation (pK_a_ tetrazole = 4–5) [1].

One of the most promising methods for losartan degradation in high-irradiance areas is solar radiation-assisted photocatalysis, which has the ability to operate with minimal fossil energy intensity. In order to improve photonic efficiency, future research should concentrate on the theoretical and experimental optimization of parabolic compound solar reactors (PCS), combining reaction kinetics and radiation transfer modeling. Simultaneously, photocatalyst immobilization on structured macroscopic supports (monoliths, foams, membranes) must be advanced in order to remove post-treatment separation stages, which are frequently overlooked in bench-scale investigations. The creation of vis-active catalysts with demonstrated hydrothermal stability, resistance to photocorrosion, and leaching of metallic dopants over extended operation in actual effluents is the main scientific problem. The constraints of quantum efficiency can be solved by integrating hybrid systems, such as solar photoelectrocatalysis combined with biological processes; however, this requires strong techno-economic validation to support municipal investments in light collecting infrastructure.

The development of decentralized and modular treatment systems is a strategic priority since a large portion of pharmaceutical pollution takes place in peri-urban or rural areas without centralized sewage networks. Adsorption in a regenerable fixed bed or low-voltage electrochemical processes are two examples of compact technologies that can be used in small communities or at points of application. However, the development of fail-safe technical architectures that necessitate little operational involvement and straightforward maintenance is essential to the sustainability of these systems. In order to ensure consistent losartan removal efficiency without requiring intricate laboratory monitoring, future research should concentrate on how resilient these systems are to random changes in flow and pollutant load, which are typical of decentralized situations. Autonomous operation and early alerts of saturation or process failure can be made possible by integrating inexpensive sensors for in situ monitoring of critical parameters (pH, conductivity, residual concentration).

The circular economy, which goes beyond purification to include the integrated recovery of resources (water, nutrients, energy, and materials), should be used to reinterpret losartan remediation. One of the future avenues involves demonstrating that loaded biosorbents (such as biochar) are safe to use as soil amendments or as substrates for bioremediation by providing a measure of the stability of the contaminants that have been retained from these materials via accelerated leaching and long-term monitoring. Similarly, the carbon cycle can be closed through the use of sophisticated oxidizing technologies to create naturally biodegradable waste products to be utilized as feedstocks in anaerobic digesters, thus enabling the transformation of environmental liabilities into energy sources. A regulatory framework based on sound science is essential for demonstrating that reintroducing treated materials or effluents to the environment does not pose an ecotoxicological risk due to the presence of losartan metabolites or unknown transformation products will require defining a safety guideline based on evidence.

The development of adequate science-based regulatory frameworks is closely linked to the practical use of remediation technology. Investment in tertiary polishing methods is currently discouraged by the lack of specified maximum permissible limits for losartan in effluents and water bodies in the majority of jurisdictions. Ecotoxicological risk-based rules that take into account the additive, synergistic, or antagonistic effects of complex combinations (cocktail effect) are anticipated to replace concentration-based regulations in the future. The adoption of cutting-edge technology will be pushed by the introduction of particular EQS for angiotensin receptor blockers and the deployment of continuous monitoring networks based on non-targeted HRMS. In addition to end-of-pipe cleanup techniques, laws that promote “green chemistry” in pharmaceutical manufacturing can lower the pollutant load at the source by using an integrated life cycle strategy. In conclusion, a transdisciplinary strategy combining materials science, process engineering, ecotoxicology, and regulatory policy is necessary for the future possibilities of losartan remediation. Developing materials with proven molecular selectivity in real matrices, optimizing solar reactors using coupled multiphysics modeling, validating decentralized systems through long-term pilot studies, establishing harmonized LCA protocols for objective technology comparison, and producing reliable ecotoxicological data to support risk-based regulations are among the top research priorities. This strategic integration will ensure that losartan technical solutions can contribute effectively to global sustainability and water security, thereby enabling the elimination of the differences between laboratory innovation and quantifiable environmental impact.

## 9. Conclusions

Consequently, effective losartan mitigation in real-world applications requires treatment trains explicitly designed to handle dynamic wastewater matrices. For instance, in municipal secondary effluents characterized by fluctuating COD (50–150 mg L^−1^) and variable natural organic matter (NOM) content, a robust tertiary hybrid configuration could integrate: (i) controlled-dose ozonation (0.5–1.0 g O_3_ g^−1^ DOC) as a primary oxidative step to cleave losartan’s biphenyl-tetrazole core and convert recalcitrant fractions into biodegradable intermediates; (ii) a BAC filter to metabolize short-chain carboxylic acids, reduce residual COD by 30–50%, and mitigate transient TP ecotoxicity; and (iii) NF as a final polishing barrier to achieve losartan concentrations <10 ng L^−1^ while ensuring pathogen removal and compliance with forthcoming EQS for angiotensin receptor blockers. To accommodate diurnal and seasonal fluctuations in influent composition, such systems should incorporate real-time UV_254_ and DOC monitoring coupled with adaptive oxidant dosing algorithms, alongside membrane fouling mitigation strategies (e.g., optimized PAC pre-coagulation or periodic air-scouring). Future research must prioritize pilot-scale validation of these adaptive hybrid trains under continuous-flow conditions, the development of losartan-specific EQS informed by integrated toxicity databases (parent + TPs), and the standardization of LC-HRMS workflows for routine environmental monitoring. Ultimately, bridging the gap between laboratory efficacy and environmental safety will require a transdisciplinary approach that couples process engineering with evidence-based regulatory frameworks, ensuring that remediation strategies for losartan are not only technically effective but also ecologically resilient and economically sustainable in complex, real-world water matrices.

## Figures and Tables

**Table 1:** Losartan physiochemical properties and environmental impact.

Parameter	Expression/Value	Environmental Implication
Molecular formula	C_22_H_23_ClN_6_O	Aromatic structure that is comparatively stable
Molar mass	422.9 g mol^−1^	Affects bioavailability and transportation
PK_a_	~4.9	Mostly ionized at room pH
log *K*_ow_	4–4.3	In neutral form, moderate hydrophobicity
log *K*_oc_	2.5–3.1	Moderate soil and sediment mobility
Solubility in water	50–100 mg L^−1^	It facilitates aqueous phase transfer.
Estimated BCF	<100	Low possibility for bioaccumulation

**Table 2:** Physiochemical properties and environmental effect compared to other ARBs.

Drug	Log *K*_ow_	pK_a_	Solubility in Water	Estimated BCF	Environmental Potential
Losartan	4–4.3	~4.9	Moderate	<100	Water mobility that is moderate
Valsartana	3.6	~4.7	Moderate	<100	Moderate perseverance
Irbesartan	2.1	~4.7	Moderate	<50	Reduced bioaccumulation
Telmisartana	7.7	~4.5	Low	>500	High bioaccumulation potential

**Table 3:** Reported losartan concentration in different aquatic environments and locations/countries.

Environmental Matrix	Concentration Range (µg L^−1^)	Region	Country	Reference
Effluent from WWTP	0.3–6.9	Copenhagen	Denmark	[8]
Effluent from a WWTP	2.4–2.5	Patancheru	India	[36]
Surface water	0.0042–1.70	Hudson river Estuary	USA	[37]
Effluent from hospitals	0.072–0.91	Coimbra	Portugal	[38]
Influent from WTP	<LOQ–1.06	Lis river		[39]
River and lake surface waters	0.003–0.0995	Valencia/Castellón	Spain	[40]
Effluent from WWTP	0.03–0.37	Castellón		
Seawater	0.0036–0.548	Guarujá beaches	Brazil	[41]
Submarine sewage	0.0007–0.0034			
Influent from WTP	0.33–0.67	Ootmarsum	The Netherlands	[42]
Effluent from WTP	0.03–0.09			
Influent from WTP	0.62–0.96	Enschede		
Effluent from WTP	0.05–0.12			
Effluent from WTP	<LOQ–0.45	Bavaria	Germany	[43]
Surface water rivers	0.12 *			
Surface waters	<LOQ–0.154	Bukovac	Serbia	[44]
Influent from WWTP	<LOQ–0.211	Catalonia	Spain	[45]
Effluent from WWTP	<LOQ–0.162			
River surface waters	<LOQ–0.821	Ter river Catalonia		
Influent from WWTP	<LOQ–0.366	Catalonia	Spain	[46]
Effluent from WWTP	<LOQ–0.075			
River surface waters	<LOQ–0.034	Onyar river Catalonia		
Seawater	0.0002–0.0086	Santo’s bay	Brazil	[25]
Surface waters	<LOQ–0.0012	Jiulong River	China	[47]

* Average.

**Table 4:** Methodological validation parameters and quality criteria for losartan present in environmental matrices.

Parameter	Typical Acceptable Range	Importance for Environmental Data
LOD	0.1–5 ng L^−1^	Evaluates the capacity to identify concentrations that are important to the environment.
LOQ	0.3–15 ng L^−1^	Establishes the minimal standard for accurate and precise quantification.
Recovery	70–120%	Assesses sample preparation effectiveness; recovery adjustments are crucial for quantitative data.
Precision (RSD)	<20% (repeatability)	Guarantees repeatability both within and between assays.
Matrix effect	±20%	The analytical signal may be suppressed or amplified by complex matrices; for correction, isotopically labeled internal standards are advised.
Selectivity	Absence of interferents in MRM transitions	Essential for preventing false positives in matrices containing hundreds of pollutants that co-occur.

**Table 5:** Removal efficiency summary and main mechanism of biological technologies for losartan removal.

Technology	Typical Removal Efficiency	Main Mechanisms	Reference
Activated sludge	20–60%	Sorption plus partial biodegradation	[68]
Aerated lagoons	10–40%	Limited biodegradation combined with dilution	[66]
MBR	40–80%	Extended biodegradation combined with membrane retention	[70]
Activated sludge with high SRT	Up to 70%	Microbial adaptation	[72]

**Table 6:** Comparison of physiochemical processes for losartan removal.

Process	Predominant Mechanism	Typical Removal Efficiency	Advantages	Limitations	Reference
Coagulation/Flocculation	Complexation, sweeping, and charge neutralization	10–40%	It eliminates turbidity and is inexpensive, well-established technology.	Sludge is produced, pH is sensitive, and poor selectivity for polar compounds.	[76,91]
Adsorption (Activated Carbon)	Electrostatic, π–π, and hydrophobic interactions	70–95%	High effectiveness, adaptability in operation, and suitability in powder or fixed bed form.	Adsorbent cost, regeneration requirements, and site competition.	[92]
Membranes (NF/RO)	Diffusion, electrostatic repulsion, and size exclusion	80–99%	Compaction, a high rejection rate, and potential process integration.	Fouling, concentrated brine production, and energy expense.	[86,87]

**Table 7:** Comparative overview of AOPS for losartan degradation.

Parameter	Ozonation	UV/H_2_O_2_	Fenton/ Photo-Fenton	Activated Persulfate
Main reactive species	OH (indirect) and O_3_ (direct)	Hydroxyl radical	Hydroxyl radical	Sulfate radical SO_4_^−^ and OH
Activation mechanism	Decomposition at alkaline pH (OH); electron transfer/cycloaddition (O_3_)	Photolysis of H_2_O_2_ (λ < 300 nm)	Fe^2+^ + H_2_O_2_ reaction; photochemical regeneration (photo-Fenton)	UV, transition metals, thermal activation, or alkaline pH
Optimal pH range	Alkaline to neutral (favors radical route)	Broad range (6–9); decreased effectiveness at high pH levels	Limit: 2.5–3.5 (traditional); solar photo-Fenton can withstand about 5	Depending on the activation technique, broad (3–11)
Efficiency in removing losartan	High (>90% in <10 min); website-specific	High; reliant on UV transmittance and H_2_O_2_ dosage	In photo-Fenton, extremely high and rapid kinetics	Maintaining high efficiency in complex matrices
Main advantages	Quick breakdown; dual action (oxidation + disinfection); absence of solid residue	UV + oxidant synergy; straightforward process; no additional metals	Inexpensive reagents High efficiency in organic matrices; solar photo-Fenton lowers energy expenses	Extended half-life radical; selectivity of electron transfer; stability of oxidizing agents
Critical limitations	O_3_’s selectivity may result in byproducts; Bromate formation and high generating energy costs	Excess H_2_O_2_ works as a scavenger; turbidity/color interference; high energy requirement (UV lights)	Ferrous sludge production; pH correction required; challenges in extracting the homogenous catalyst	Persulfate is more expensive than H_2_O_2_; salinization (sulfate ions) is a risk; thermal activation uses energy
Formation of by-products	Danger of short-chain aldehydes, ketones, and bromates (if Br^−^ is present)	Oxalic and formic carboxylic acids; potential production of halogenated byproducts in halide matrices	Hydroxylated aromatic intermediates and short-chain carboxylic acids; incomplete mineralization may lead to iron-complexed organic species that require monitoring.	Sulfonated byproducts; potential persistence of intermediates because of SO_4_^−^
Estimated energy consumption	High (generation of O_3_ on-site)	High (UV systems with medium or low pressure)	Low (solar); Moderate (photo-Fenton with lighting); Low (traditional Fenton)	Moderate to High (method-dependent: heat > UV > chemical)
Relative operating cost	Elevated	From moderate to high	Moderate (with sludge treatment) to low (reagents)	From moderate to high
Toxicity aspects of TPs	Temporary increases in acute toxicity may require biological post-treatment.	More polar and bioavailable TPs may result from incomplete mineralization	Sludge can concentrate adsorbed metals and TPs, just like other AOPs	Monitoring is crucial due to the potential production of sulfonated TPs with poorly understood toxicity
Real-scale applicability	Developed in cutting-edge wastewater treatment facilities in Europe; requires stringent bromate control	Energy costs at high flow rates limit its use in tertiary polishing.	Suitable for concentrated industrial effluents; municipal sludge management issues	Emerging; needs economic feasibility studies; promise for stubborn effluents

**Table 8:** Comparative overview of heterogeneous photocatalytic systems for losartan degradation.

Parameter	TiO_2_ (Pure)	Doped Materials (TiO_2_-X)	Carbon-Based Composites (TiO_2_/C)
Typical structure/phase	Anatase, Rutile, or mixed-phase (e.g., P25)	N, C, S, Fe, Ag, and Cu doping of TiO_2_	TiO_2_ supported on carbon nanofibers, graphene, carbon nanotubes, or charcoal
Energy bandgap	~3.2 eV (anatase); ~3 eV (rutile)	Diminished: 2.5–3 eV, depending on the dopant	Like pure TiO_2_, but with easier charge transfer
Spectral activation range	UV (λ < 387 nm)	Extended UV-Vis (for N doping, up to about 550 nm)	Extended to
Losartan degradation mechanism	Attack on the imidazole/tetrazole ring; production of e^−^/h^+^; creation of •OH and O_2_^−^	Dopants can produce more active sites using the same method, but with increased absorption of visible photons	Losartan’s prior adsorption onto carbon and electron transfer to TiO_2_; adsorption–oxidation synergy
Removal efficiency	High in artificial UV (>90% in 60–120 min); moderate in direct sunshine	Depending on dopant stability, moderate to high in sunlight (60–95% in 60–180 min)	High (85–98% in 30–90 min); local pollutant concentration increases efficiency
Mineralization (removal of color)	Moderate (40–70%); persistent recalcitrant intermediates	Variable (30–80%); byproducts may be released by unstable doping agents	High (60–90%); the carbon structure promotes the intermediates’ progressive oxidation
Main advantages	Chemical and mechanical stability; inexpensive and widely accessible; non-toxic and well-characterized	Activation in the presence of visible or solar light; Possibility of increased quantum efficiency; Dopant versatility	Improved separation of charges; High capacity for adsorption; potential application of biochar (circular economy)
Critical limitations	UV-only spectrum limitation; Quick electron/hydrogen ion recombination; challenging recovery in slurry systems	Metallic dopant leaching; photo corrosion and deactivation over time; More expensive and intricate synthesis	Expensive graphene/CNTs; difficulties recovering and separating the nanocomposite; Nanomaterial leaching risk
Formation of by-products	Aldehydes, carboxylic acids (oxalic, formic), and potential temporary toxicity	Dopants can catalyze several oxidation routes, just like pure TiO_2_	Decreased intermediate buildup as a result of successive oxidation; sulfonated or nitrogenated TPs must be monitored
Stability/Reusability	High (more than five cycles without a discernible drop-in activity)	Moderate (3–5 cycles); activity loss as a result of dopant leaching or sintering	Variable: graphene/CNTs may experience mechanical deterioration; biochar shows good stability
Estimated energy consumption	Low under direct solar radiation (although with reduced efficiency); high if reliant on artificial UV	Moderate; using more of the sun’s visible portion lessens the need for UV supplementing	Moderate; comparable to doped medications; adsorption–oxidation synergy may shorten response times
Relative cost of synthesis	Low (widely accessible commercial material)	Moderate to high (depending on the inclusion technique and doping agent)	High for graphene/CNTs; low to moderate for biochar made from waste
Real-scale applicability	Developed in solar pilot systems; immobilization problems to prevent catalyst loss	Emerging; needs validation of long-term stability in complicated matrices	The engineering problems for recovering and containing nanoparticles seem promising, particularly with biochar
Aspects of toxicity/ecotoxicity	TPs may exhibit transient acute toxicity; testing with *Daphnia* and *Vibrio* is recommended	Extra danger of toxicity due to metal leakage; crucial TP characterization	Risk of nanomaterial leaching; a comprehensive ecotoxicological effect evaluation is required

**Table 9:** Technical comparison of key features and performance indicators for the electrochemical AOPS for losartan removal.

Parameter	Electro-Oxidation	Electro-Fenton
Main reactive species	Indirect oxidants (Cl_2_, O_3_, and H_2_O_2_) produced in situ; •OH adsorbed on the anode	Fenton reaction (Fe^2+^; H_2_O_2_) produces hydroxyl radical (•OH) in solution
Mechanism of oxidant generation	Indirect oxidation caused by electrogenerated species or direct oxidation at the anode surface	Electrochemical regeneration of Fe^2+^ from Fe^3+^ plus cathodic reduction of O_2_ to H_2_O_2_
Typical electrode materials	BDD, PbO_2_, DSA (Ti/RuO_2_-IrO_2_), and SnO_2_ are anodes; stainless steel, graphite, and glassy carbon are cathodes	Porous carbon, carbon felt, and gas-diffusion electrodes (GDEs) are cathodes; BDD, Pt, and DSA^®^ are anodes; Fe^2+^ is added or produced anodically
Optimal pH range	Broad (3–10); reliant on the matrix and the method (direct or indirect)	Restricted: 2.5–3.5 (to enhance •OH production and prevent Fe^3+^ precipitation)
Efficiency in removing losartan	High (>90% in 30–90 min with BDD); reliant on conductivity and current density	Very high (>95% in 20–60 min); kinetics enhanced by continuous bulk OH production
Mineralization (removal of color)	BDD prefers full mineralization; moderate to high (50–90%)	High (60–95%); attack on resistant intermediates is favored by oxidation in bulk solution
Main advantages	Exact management of applied potential; no ongoing reagent addition; room temperature functioning and modularity; High mineralizing capacity is provided by BDD	High efficiency in complicated matrices; electrochemical regeneration of Fe^2+^ lowers iron consumption and sludge production; in situ H_2_O_2_ generation eliminates storage and transportation
Critical limitations	High energy consumption for full mineralization; formation of halogenated byproducts in matrices containing Cl^−^; high cost of BDD; and electrode surface fouling	Iron precipitation at neutral pH; sensitivity of H_2_O_2_ production to the matrix (radical scavengers); operational modifications are necessary for acidic pH restriction; Optimization of parameters (current, O_2_ flow rate, [Fe^2+^]) is complicated
Formation of by-products	Risk of short-chain carboxylic acids as final intermediates, bromates (if halides are present), and organochlorines	Like EO, hydroxylated intermediates may continue if mineralization is not complete; iron-organic complexes may occur
Estimated energy consumption	Moderate to High (5–50 kWh m^−3^ or 10–100 kWh kg^−1^ COD); based on the electrode’s conductivity and composition	Moderate (3–30 kWh m^−3^); effective H_2_O_2_ cathodes and Fe^2+^ regeneration improve energy efficiency
Relative operating cost	High (particularly with BDD); moderate with PbO_2_ or DSA	High (particularly with BDD); moderate with PbO_2_ or DSA
System stability/reusability	High for stable anodes (BDD, DSA); fouling can necessitate routine replacement or cleaning	Moderate: Over time, iron precipitation and porous cathode degradation can lower efficiency
Real-scale applicability	Cost issues for high flow rates; consolidated for small and medium-sized industrial effluents	Pilot-scale emergence; potential for high-organic-load concentrated effluents; needs long-term validation
Aspects of toxicity/Ecotoxicity	Halogenated byproducts have the potential to increase toxicity	Iron-TP complex monitoring is crucial because transient toxicity peaks in intermediate stages

**Table 10:** Technical comparison of features and application of novel adsorbent material for losartan removal.

Parameter	Biochar	Graphene-Based Materials	MOFs	Functional Biopolymers	Nanomaterials
Typical composition/structure	Porous carbon produced by pyrolyzing biomass (lignocellulose, agricultural waste)	GO, rGO, and hybrid materials	Metal ions (Zn, Zr, and Fe) and organic ligands (imidazolates, carboxylates) form porous crystalline formations	Cross-linked natural polymers, modified cellulose, chitosan, and alginate	Mesoporous silica (SBA-15, MCM-41), magnetic nanoparticles (Fe_3_O_4_), nZVI, CNTs (single/multiple walls), and hybrid nanocomposites
Specific surface area	Moderate: 50–400 m^2^ g^−1^ (with chemical activation, it can reach >800 m^2^ g^−1^)	Extremely high: 300–2600 m^2^ g^−1^ (depending on the level of functionalization and reduction)	Outstanding: 500–7000 m^2^ g^−1^ (ultra-high porosity and tunable)	Low to moderate: 10–150 m^2^ g^−1^ (increases with porosity generated by freeze-drying)	Extremely high: 200–1500 m^2^ g^−1^ (mesoporous silica: 600–1000 m^2^ g^−1^; CNTs: 400–1200 m^2^ g^−1^)
Losartan adsorption mechanisms	Pore filling, hydrophobic interactions, hydrogen bonding, and π–π stacking	Electrostatic interactions, complexation with oxygenation groups, and π–π stacking dominance	Van der Waals and electrostatic interactions, metal-ligand coordination, and spatial constriction in pores	Ionic complexation, hydrogen bonding, and electrostatic interactions (amine/carboxyl)	Adsorption in meso/microporous pores, surface complexation, electrostatic interactions, π–π stacking (CNTs), and magnetic interactions (for separation)
Reported adsorption capacity	Moderate: 20–150 mg g^−1^ (depending on source material and activation)	High: 100–350 mg g^−1^ (interaction with aromatic rings is favored by the π-rich surface)	Extremely high: 150–500 mg g^−1^ (porosity intended for molecular recognition)	15–120 mg g^−1^ is low to moderate (increases with chemical functionalization)	High to extremely high: 80–450 mg g^−1^ (functionalized carbon nanotubes: 150–300 mg g^−1^; magnetic nanocomposites: 100–400 mg g^−1^)
Optimal pH range	Broad range (4–9); decreased effectiveness at high pH levels because of surface charge variations	5–8; high ionic strength aggregation may make active sites less accessible	Limited (4–7); a lot of MOFs hydrolyze at basic or acidic pH levels	Depending on the polymer’s pKa: alginate (pH 5–7), chitosan (pH 4–6)	Broad-range stable magnetic nanoparticles; variable: 3–9 (depending on surface functionalization)
Adsorption kinetics	Moderate: equilibrium in 30 to 180 min; rate may be limited by diffusion through holes	Quick: strong diffusivity on two-dimensional surfaces; equilibrium in 10–60 min	Diffusion through micropores may be limiting; variable: 15–120 min	Slow to moderate: 60–240 min; diffusion may be delayed by polymer swelling	Fast to extremely fast: 5–45 min (high diffusivity in nanostructures; process accelerated by magnetic separation)
Main advantages	low expense and little waste; Circular economics and sustainability; chemical stability over a broad pH range	Remarkable adsorption capability; surface that can be chemically functionalized; Conductivity of electricity in hybrid applications	High capacity and particular affinity; adaptable porosity and selectivity; and synthesis versatility	Low toxicity and biodegradability; Raw material abundance and chemical functionalization ease	High surface area and faster kinetics; magnetic separation facilitation; Adaptability for selection and potential use in continuous systems
Critical limitations	Standardization is hampered by structural variation; pollutants could seep from biomass; Quick saturation in intricate matrices	High cost of processes graphene of superior quality; Risk of nanosheet leaching; propensity to agglomerate in saline solutions	Poor hydrothermal stability in water; metal ion release during hydrolysis; Complex synthesis using organic solvents	Degradation during regeneration cycles; reduced capacity compared to carbonaceous materials; excessive swelling and poor mechanical stability	Danger of nanoparticles seeping into the wastewater; expensive controlled synthesis; combining elements in complicated matrices; Inadequately described nanoparticle ecotoxicity
Regeneration potential	Moderate: capacity reduction after three to five cycles; thermal or organic solvent desorption	Moderate to high: graphene shows good structural stability after washing with ethanol and NaOH	Limited: chemical regeneration may deteriorate the structure; many MOFs break upon desorption	Low: crosslinking may hinder full desorption; biopolymers break down with subsequent cycles	Moderate: Stable functionalization’s support 4–8 cycles; aggregation may decrease efficiency; magnetic nanoparticles make separation simple
Relative cost of synthesis	Low (simple pyrolysis; waste as raw material)	High (controlled procedures and costly reagents are needed for graphene synthesis)	High to extremely high (controlled synthesis conditions, synthesized organic ligands)	Low to moderate (plenty of raw materials; cost increases due to functionalization)	Moderate to high (Magnetic nanoparticles: moderate cost with scalability; CNTs and mesoporous silica: controlled synthesis)
Environmental risks/Ecotoxicity	Leaching of metals, phenols, or PAHs from the initial biomass; saturated biochar disposal needs to be managed	Release of carbon nanoparticles with unclear ecotoxicity; difficulties in separation	Leaching of organic ligands and metal ions (Zn^2+^, Zr^2+^); residues are produced via hydrolytic degradation	Low intrinsic toxicity, although glutaraldehyde and other crosslinking agents may be cytotoxic	Unintentional discharge of potentially ecotoxic nanoparticles (oxidative stress in aquatic species); CNTs’ environmental persistence; and the necessity of control measures
Real-scale applicability	Potential for decentralized systems; difficulties with continuous flow recovery and standardization	Applications at high flow rates are limited by the emergence of nanomaterial separation and associated costs	Laboratory/pilot stage; expenses and water stability hinder industrial scale-up	Tertiary polishing is appropriate; immobilization on supports is necessary due to mechanical restrictions	Emerging with great potential: cost and regulatory issues with nanoparticles in wastewater; magnetic separation enables continuous operation

## Data Availability

Where no new data were created.

## Abbreviations

The following abbreviations are used in this manuscript:

AC	Activated Carbon
ACNF	Acid-treated Carbon Nanofibers
AOP	Advanced Oxidation Process(es)
ARB	Angiotensin II Receptor Blocker(s)
BCF	Bioconcentration Factor
BDD	Boron-Doped Diamond
BMOF	Bifunctional Metal–Organic Framework
CAS	Conventional Activated Sludge
CNT	Carbon Nanotubes
COD	Chemical Oxygen Demand
CYP2C9	Cytochrome P450 2C9
DSA	Dimensionally Stable Anode
dSPE	Dispersive Solid-Phase Extraction
EC_50_	Effective Concentration (50%)
EF	Electro-Fenton
EQS	Environmental Quality Standards
EXP-3174	Losartan carboxylic acid metabolite
EXP-3179	Losartan carboxaldehyde metabolite
GDE	Gas Diffusion Electrode
GO	Graphene Oxide
HRMS	High-Resolution Mass Spectrometry
LC-MS/MS	Liquid Chromatography–Tandem Mass Spectrometry
LCA	Life Cycle Assessment
LOD	Limit of Detection
LOQ	Limit of Quantification
MF	Microfiltration
MOF	Metal–Organic Framework
MRM	Multiple Reaction Monitoring
NC	Nanocellulose
NF	Nanofiltration
PEC	Predicted Environmental Concentration
PEF	Photoelectro-Fenton
PNEC	Predicted No-Effect Concentration
QqQ	Triple Quadrupole
rGO	Reduced Graphene Oxide
RO	Reverse Osmosis
RQ	Risk Quotient
SPE	Solid-Phase Extraction
SRT	Solids Retention Time
STP	Sewage Treatment Plant
TOC	Total Organic Carbon
TP	Transformation Product
UF	Ultrafiltration
UHPLC-MS/MS	Ultra-High-Performance Liquid Chromatography–Tandem Mass Spectrometry
UV	Ultraviolet
UV-C	Ultraviolet C
WWTP	Wastewater Treatment Plant(s)
